# Efficacy of neurosurgical intervention in syrinx resolution in patients presenting with Chiari malformation type I and syringomyelia: a systematic review and radiological meta-analysis

**DOI:** 10.1007/s10143-025-03864-9

**Published:** 2025-10-20

**Authors:** Adharsh Suraj Prasad, Aqif Farhan bin Azmil Farid, Isaac Tang Jing Wen, Thomas Zhang, Chandrasekaran Kaliaperumal

**Affiliations:** 1 University of Edinburgh Medical School, Chancellor’s Building, 49 Little France Crescent, Edinburgh, Scotland, EH16 4SB UK; 2 Aberdeen Royal Infirmary, Aberdeen, Scotland, AB25 2ZN UK; 3 Royal Infirmary Edinburgh, Edinburgh, Scotland, EH16 4SA UK; 4 Department of Neurosurgery, Department of Clinical Neurosciences, 50 Little France Crescent, Edinburgh, Scotland, EH16 4TJ UK

**Keywords:** Chiari malformation, Syringomyelia, Posterior fossa decompression

## Abstract

Neurosurgical techniques have been used to treat Chiari Malformation type 1 (CM1). However, there remains some uncertainty regarding which neurosurgical procedure is most efficacious in treating patients with CMI and syringomyelia. Our study aims to compare the efficacy of available neurosurgical techniques in resolving syrinx for patients with CM1 and syringomyelia. Included studies must report both pre-and post-operative syrinx measurements, discuss the use of nurosurgery, and involve patients with CM1 and syringomyelia. Studies with less than 5 patients were excluded. Databases used to identify studies included PubMed, Scopus, Cochrane Library, Web of Science, and Ovid. The Newcastle-Ottawa Scale, Joanna Briggs Institute checklist, and the Risk of Bias 2 analysis tools assesed the risk of bias in the study. A meta-analysis was conducted using a random-effects model, and forest plots illustrated the results. A total of 20 studies involving 3,063 patients with CM1 and syringomyelia were included in the study. Posterior fossa decompression with duraplasty (PFDD) demonstrated significant syrinx reduction: syrinx width (SW) reduced by a mean difference (MD) of 2.46 mm (95% CI: 2.16–2.76), syrinx length by 1.68 vertebral segments (95% CI: 1.44–1.92), and syrinx-to-cord ratio by 0.21( 95% CI: 0.15 to 0.26). Posterior fossa decompression with tonsillar reduction (PFDTR) had significantly reduced SW (MD: 2.85 mm, 95% CI: 1.99–3.71). It was superior to PFDD in the syrinx-to-cord ratio (MD: 0.04, 95% confidence interval (CI) of 0.01 to 0.08). Fourth ventricular stents (FVS) and syringo-subarachnoid shunts (SSS) were other reported techniques, but there were insufficient studies for formal analysis. PFDD and PFDRT are both efficacious in reducing syrinx size in patients with CM1 and syringomyelia. PDRT displayed modest superiority in syrinx resolution, and the clinical significance of this is limited. More studies are needed to determine the efficacy of FVS and SSS.

**Supplementary Information:**

The online version contains supplementary material available at 10.1007/s10143-025-03864-9.

## Introduction

Chiari malformation encompasses a spectrum of congenital hindbrain abnormalities affecting the cerebellum, skull base, brainstem, and cervical spinal cord. Chiari Malformation Type 1 (CM1) is the most common subtype, with an estimated prevalence of approximately 1% in the paediatric population [1]. It is characterised by downward displacement of the cerebellar tonsils through the foramen magnum, leading to compression of neural structures and disruption of cerebrospinal fluid (CSF) flow. This anatomical disturbance is frequently associated with syringomyelia—a fluid-filled cavity (syrinx) within the spinal cord, which occurs in 50–75% of Chiari malformation type 1 (CM1) cases [2].

Individuals diagnosed with CM1 with syringomyelia may present with occipital or cervical pain, coordination difficulties, facial muscle weakness, and autonomic dysfunction. Diagnosis is primarily established through magnetic resonance imaging (MRI), sometimes supplemented with CSF flow studies to assess dynamic obstruction.

Management of CM1 with syringomyelia includes both conservative and surgical approaches. Surgical intervention aims to restore normal CSF dynamics and reduce the size of the syrinx. The most widely used surgical procedure is posterior fossa decompression. However, there are several variations of this technique, including posterior fossa decompression with osseous-only decompression (PFD), posterior fossa decompression with duraplasty (PFDD), and posterior fossa decompression with tonsillar manipulation (PFDRT). The optimal surgical technique used has been a longstanding and heavily debated topic within the literature, with some favouring PFD [3] because it has fewer complications, while others favour PFDD [4] or PFDRT [5] because they report better clinical outcomes. Other techniques used for treating syringomyelia in CM1 include fourth ventricle stents [6] and shunts [7], though their comparative effectiveness remains unclear.

Existing systematic reviews have evaluated surgical outcomes in CM1 + syringomyelia using radiological outcomes; however, most of these reviews focus on subjective categorisation, such as ‘syrinx resolution,’ and do not report quantifiable changes in syrinx dimension (e.g., width, length, and syrinx-to-cord ratio). Most reviews also primarily focus on investigating the comparative efficacy between posterior fossa decompression with and without duraplasty, with little attention given to other existing neurosurgical techniques, such as fourth ventricular stenting. Comparing available neurosurgical techniques could inform clinical management of strategies for this complex condition. Additionally, analysing quantitative MRI-based measurements pre- and post-operatively could provide a more consistent and reproducible means of assessing treatment efficacy, particularly in guiding surgical decision-making.

Therefore, we aim to identify which neurosurgical techniques are most effective in reducing or resolving syrinx size. To do this, we conducted a systematic review of the current literature on surgical interventions for CM1 associated with syringomyelia, with a specific focus on radiological measures of syrinx. To our knowledge, this would be the first review to systematically synthesise and analyse continuous radiological measurements of syringomyelia pre- and post-operatively for a variety of neurosurgical techniques, which would allow for a more in-depth understanding of surgical efficacy.

## Methodology

### Inclusion & exclusion criteria

This study was conducted in accordance with the Preferred Reporting Items for Systematic Reviews and Meta-Analyses (PRISMA) guidelines. The protocol for this study was registered on PROSPERO under the ID CRD42024576709. Our study includes both non-randomised and randomised studies, and no limitations were imposed on the age of the patients included. However, for studies to be included in our review, they must consist of patients diagnosed with CM1 and syringomyelia, discuss the use of neurosurgical intervention, and be published in English. Additionally, studies must also provide metrics before and after surgery. Case reports, editorials, letters, reviews, animal studies, and studies with a sample size of less than five were excluded from our studies.

### Search strategy

A formal search for studies was conducted on PubMed, Scopus, Cochrane Library, Web of Science and Ovid. Search terms used include keywords such as “Chiari Malformation Type I,” “neurosurgery,” and “syringomyelia.” MeSH terms, truncation, and title/abstract screening were also incorporated into our search strategy to ensure appropriate identification of studies, and complete details of the search strategy can be found within the *supplementary material.* Two independent reviewers conducted full-text screening and data collection, and a third reviewer resolved differences.

### Data extraction

The primary outcomes sought included any radiological measures of syrinx size, such as syrinx width, syrinx length, syrinx area, syrinx volume, and the syrinx-to-cord (S/C) ratio, both preoperatively and post-operatively. Other outcomes included in our study are title, author, population size, percentage male, mean age, number of patients with syrinx resolution, number of patients with worsened syrinx, preoperative hydrocephalus, scoliosis, and Chicago Chiari Outcome Scale (CCOS) scores. Where applicable, outcomes such as the number of patients with hydrocephalus or scoliosis must be specific to patients with CM1 and syringomyelia. In studies that included both CM1 patients with and without syringomyelia, if data were not explicitly specified for the CM1 and syringomyelia population group, the data were recorded as “n/a”. We also applied the same method to demographic variables, such as patient age and sex distribution, for which the original study did not explicitly report values. These studies were still included in our analysis to avoid selection bias; however, missing fields were reported as “n/a”, and no imputation was performed.

### Assessment for risk of bias

Risk of bias was assessed using the Newcastle-Ottawa scale (NOS) [8] for observational studies, the Cochrane Risk of Bias 2 (Rob2) tool [9] for randomised controlled trials, and the Joanna Briggs Institute (JBI) checklist for case series [10]. Two independent reviewers assessed the risk of bias, and differences were resolved through discussion. Studies assessed with NOS were categorised into low (0–3), moderate (4–6), and high quality (7–9). Likewise, for the JBI checklist, studies would be classified into low (0–5), moderate (6–7), and high quality (8–10).

### Statistical analysis

Subgroup meta-analyses on specific neurosurgical techniques were conducted if outcomes were reported by at least three studies included in our final analysis. Hence, studies reporting SSS and FVS were only included in our descriptive synthesis and excluded from our meta-analysis, as each surgical technique was represented by only one study. Studies must also employ the same methodologies (e.g., cohort study, randomised controlled trial), populations (i.e., reoperation, first surgery), outcomes, and measures of uncertainty. Raw data were used to calculate effect sizes when they were not reported in individual studies. For continuous outcomes, such as syrinx width and length, the mean difference was calculated using pre- and post-operative means and standard deviation. The standard deviations for mean differences were estimated using an assumed correlation coefficient of 0.5 (Cochrane Handbook) [11]. A sensitivity analysis was conducted with *r* = 0.3 and *r* = 0.7. Across all our analyses, this yielded stable pooled estimates, indicating that our results remain robust to the choice of correlation coefficient. For dichotomous outcomes, we calculated event proportions. From these calculations, we generated pooled estimates and 95% confidence intervals using a random-effects meta-analysis model in R Studio. Forest plots were synthesised for each subgroup meta-analysis conducted. I2 statistic, Cochrane’s Q test, and t2 estimate were used to identify statistical heterogeneity between studies.

Reporting bias was visually assessed using a funnel plot for asymmetry. An Egger’s test was conducted, which included at least 10 studies in the analysis. It was omitted when fewer studies were available, as per the Cochrane Handbook [11]. If fewer than 10 studies were included in the analysis, the funnel plot would be used for visual inspection alone (*Supplementary figures*).

## Results

### Search & selection

**Fig. 1:**
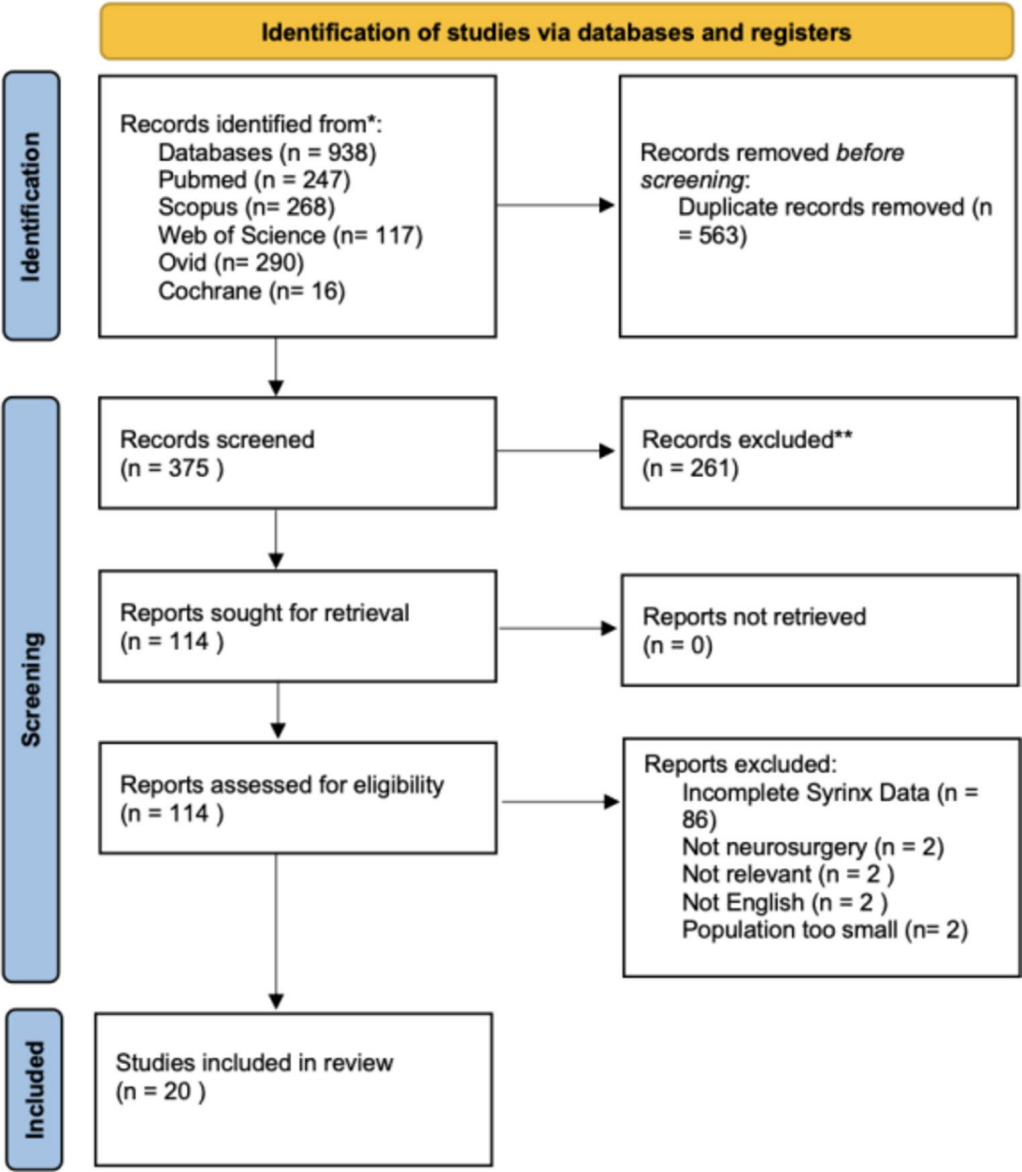
PRISMA Flowchart

The result from our search is highlighted in our PRISMA flow diagram (Fig. 1). A total of 983 records were identified through the databases, of which 20 were included [12–31] in our final analysis. There were no studies that met our inclusion criteria and were excluded. Common reasons for exclusion during the full-text screen included an incomplete report of syrinx measurements pre-and post-operatively, as well as a sample population that was too small.

### Risk of bias

The NOS was conducted on all selected cohort studies, and a score of 7 or higher indicated a study of high quality. Of the 20 studies selected for the final analysis, 17 were cohort studies, of which 14 were of high quality, and three were of moderate quality (each scoring 6) (Table 1). Our study also included 2 case series by Han et al. [20] and Geng et al. [15]. Both studies met at least 8 out of 10 criteria for the JBI Critical Appraisal tool for case series (9 and 8, respectively) and were deemed to be of high quality (Supplementary Fig. 1). The study by Jiang et al. [27] was the only randomised controlled trial selected for our final analysis; however, there was a suggestion of high risk of bias in the third domain following analysis using the RoB2 tool (Supplementary Fig. 2).

**Table 1:**
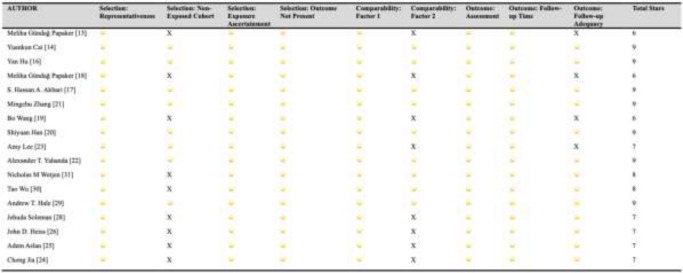
Summary of NOS assessment for cohort studies

### Demographics

Our study included a total of 3,063 patients presenting with CM1 and syringomyelia. The percentage of males for this population was difficult to determine, as not all studies reported sex differences specific to patients with CM1 + syringomyelia. Details for demographics specific to the overall population and those specific to patients with CM1 and syringomyelia are highlighted in Tables 2 and 3, respectively.

**Table 2:**
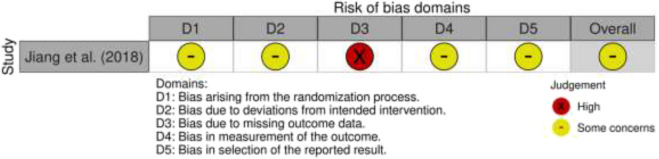
Summary of RoB 2 analysis

**Table 3:** Summary of JBI checklist for case series

Author	Were there clear criteria for inclusion in the case series?	Was the condition measured in a standard, reliable way for all participants included in the case series?	Were valid methods used for identification of the condition for all participants included in the case series?	Did the case series have consecutive inclusion of participants?	Did the case series have complete inclusion of participants?	Was there clear reporting of the demographics of the participants in the study?	Was there clear reporting of clinical information of the participants?	Were the outcomes or follow up results of cases clearly reported?	Was there clear reporting of the presenting site(s)/clinic(s) demographic information?	Was statistical analysis appropriate?
Han et al. [12]	Yes	Yes	Yes	Unclear	Yes	Yes	Yes	Yes	Yes	Yes
Geng et al. [15]	Yes	Yes	Yes	Unclear	Unclear	Yes	Yes	Yes	Yes	Yes

### Neurosurgical techniques

Several neurosurgical techniques were reported in our study. Most of these studies could be classified into one of the three categories: PFD, PFDD, and PFDRT. Other techniques reported in the final 20 studies that could not be categorised were FVS after PFD, posterior fossa decompression with duraplasty and obex exploration (PFDDO), posterior fossa decompression with dura-splitting (PFDS), posterior fossa decompression with superficial durotomy (PFDSD) and SSS. The most common procedure reported was PFDD.

### Radiological and clinical outcomes

Radiological outcomes, including syrinx width, syrinx length, syrinx area, syrinx volume, and S/C ratios, are reported in Table 4 for all included **studies.** Other outcomes, such as the number of syrinx resolutions and worsened syrinx size after surgical intervention, are reported in Table 5. The most common radiological measure was syrinx width. In contrast, other measures, such as syrinx volume and area, were reported by only two studies. For all neurosurgical techniques identified, positive outcomes on radiological syrinx measures were measured. However, a meta-analysis could not be conducted for all, as some techniques were insufficiently reported within the final set of studies included (e.g. FVS and SSS) (Tables 6 and 7).

**Table 4:** Study details and demographics of entire cohort

Paper	Author	Country	Study period	Study type	Cohort size	Average age of entire cohort	%Male of entire cohort
Fourth ventricular suabarachnoid stent for Chiari malformation type I–associated persistent syringomyelia	Han et al. (2023) [12]	USA	2009–2022	Retrospective case series	13	28.7 (5–67)	23.1
Surgical Outcomes of Adult Chiari Malformation Type 1: Experience at a Tertiary Institute	Gündağ Papaker et al. (2021) [13]	Turkey	2012–2017	Retrospective cohort study	25	36.6±11.4	16
Prognostic analysis of posterior fossa decompression with or without cerebellar tonsillectomy for Chiari malformation type I	Cai et al. (2023) [14]	China	2016–2021	Multicenter retrospective study	125	PFDD (47.26 ± 8.88) or PFDRT (46.22 ± 7.562)	PFDD: 25.6; PFDRT 75.6
Dura-splitting versus a combined technique for Chiari malformation type I complicated with syringomyelia	Geng et al. (2018) [15]	China	2008–2016	Retrospective comparative case series	49	PFDS: 39.00 ± 10.85, PFDRT: 42.13 ± 9.15	PFDS: 35.3; PFDRT :21.9
A long-term follow-up study of adults with Chiari malformation type I combined with syringomyelia	Hu et al. (2023) [16]	China	2013–2020	Retrospective cohort study	158	PFDRT: 47.97 ± 10.83; PFDD: 46.14 ± 10.75	PFDRT: 23.5; PFDD: 31.1
Complications and outcomes of posterior fossa decompression with duraplasty versus without duraplasty for pediatric patients with Chiari malformation type I and syringomyelia: a study from the Park-Reeves Syringomyelia Research Consortium	Akbari et al. (2022) [17]	USA	2001–2018	Retrospective + prospective multicenter cohort	692	PFD: 9.46 ± 4.14, PFDD: 9.94 ± 4.64	PFD: 46.2; PFDD: 39.0
Clinical and radiological evaluation of treated Chiari I adult patients	Papaker et al. (2021) [18]	Turkey	2010–2017	Retrospective cohort (2 centers)	72	38.0 ± 11.7	27.8
Long-term outcomes of foramen magnum decompression with duraplasty for Chiari malformation type I in adults	Wang et al. (2023) [19]	China	2011–2020	Retrospective cohort	297	43.08 ± 10.16	22.9
Individualized Functional Decompression Options for Adult Chiari Malformation With Syringomyelia and A Novel Scale	Han et al. (2023) [20]	China	2014–2022	Retrospective single-center cohort	78	43.5 ± 16.25	23.1
Exploring the prognostic differences in patients of Chiari malformation type I with syringomyelia undergoing different surgical methods	Zhang et al. (2023) [21]	China	2016–2021	Retrospective single-center cohort	182	46.39 ± 10.58	28.4
Dural augmentation approaches and complication rates after posterior fossa decompression for Chiari I malformation and syringomyelia	Yahanda et al. (2021) [22]	USA	2001–2019	Retrospective multicenter cohort (PRSRC database)	781	Autograft: 10.3 ± 4.6Nonautologous: 10.2 ± 4.7	Autograft: 42.3Nonautologous: 41.5
Comparison of posterior fossa decompression with or without duraplasty in children with Type I Chiari malformation	Lee et al. (2014) [23]	USA	2003–2011	Retrospective single-center cohort	65	PFDD: 9.9 ± 5.3PFD: 8.9 ± 5.2	PFDD: 44PFD: 52
Comparison decompression by duraplasty or cerebellar tonsillectomy for Chiari malformation-I complicated with syringomyelia	Jia et al. (2019) [24]	China	2013–2016	Retrospective single-center cohort	115	PFDRT: 43.59 ± 10.44PFDD: 42.92 ± 11.66	PFDRT: 26.9PFDD: 37.8
Posterior Fossa Decompression and superficial durotomy rather than complete durotomy and duraplasty in the management of Chiari 1	Aslan et al. (2021) [25]	Turkey	2012–2019	Retrospective cohort (single center)	54	37.51 ± 15.14	33.3
Pathophysiology of persistent syringomyelia after decompressive craniocervical surgery: Clinical article	Heiss et al. (2010) [26]	USA	n/a	Prospective clinical cohort study	16	35 ± 14	n/a
Comparison of Clinical and Radiographic Outcomes for Posterior Fossa Decompression with and without Duraplasty for Treatment of Pediatric Chiari I Malformation	Jiang et al. (2018) [27]	China	2011–2015	Prospective randomized controlled trial	82	PFD: 13.64 (10–18)PFDD: 13.95 (10–18)	PFD: 50.0PFDD: 50.0
Syringo-Subarachnoid Shunt for the Treatment of Persistent Syringomyelia Following Decompression for Chiari Type I Malformation	Soleman et al. (2017) [28]	Israel/Switzerland	2003–2016	Retrospective cohort	21	16.3 ± 15.4	33.3
Factors associated with syrinx size in pediatric patients treated for Chiari malformation type I and syringomyelia	Hale et al. (2020) [29]	USA	2001–2015	Retrospective multi-center cohort (PRSRC registry)	380	10.05 ± 4.40	40
Syrinx resolution after posterior fossa decompression in patients with scoliosis secondary to Chiari malformation type I	Wu et al. (2012) [30]	China	2000–2009	Retrospective single-center cohort	44	12.1 years (range 6–18)	59.1
Time course of syringomyelia resolution following decompression of Chiari malformation Type I	Wetjen et al. (2008) [31]	USA	n/a	Prospective cohort (NIH Clinical Center)	29	37 ± 12 years (range 16–61)	27.6

**Table 5:** Demographics for CM1 + syringomyelia

Author	Neurosurgical procedure	Number of CM1 with syrinx	Average age (CM1 + syrinx)/years	% Male (CM1+syrinx)	Hydrocephalus (pre-op)	Scoliosis
Han et al. (2023) [12]	FVSS (reoperation)	13	28.7 (5–67)	23.1	n/a	1
Gündağ Papaker et al. (2021) [13]	PFDD	8	n/a	n/a	0	n/a
Cai et al. (2023) [14]	PFDD and PFDRT	70 [PFDD (n=47) and PFDRT (n=23)]	n/a	n/a	PFDD: 2; PFDRT: 2	n/a
Geng et al. (2018) [15]	PFDS and PFDRT	49	Dura: 39.00 ± 10.85, Comb: 42.13 ± 9.15	Dura: 35.3; Comb: 21.9	0	n/a
Hu et al. (2023) [16]	PFDRT or PFDD	158 [PFDRT (68) and PFDD (90)]	PFDRT: 47.97 ± 10.83; PFDD: 46.14 ± 10.75	PFDRT: 23.5; PFDD: 31.1	0	n/a
Akbari et al. (2022) [17]	PFDD and PFDD	692 [PFD (n=117) vs PFDD (n=575)]	PFD: 9.46 ± 4.14, PFDD: 9.94 ± 4.64	PFD: 46.2; PFDD: 39.0	n/a	n/a
Papaker et al. (2021) [18]	PFDD	23	n/a	n/a	n/a	n/a
Wang et al. (2023) [19]	PFDD	265	n/a	n/a	n/a	n/a
Han et al. (2023) [20]	PFD, PFDD, and PFDDO	78 [PFD (n=22), PFDD (n=20), PFDDO (n=36)]	43.5 ± 16.25	23.1	n/a	0
Zhang et al. (2023) [21]	PFDRT and PFDD	182 [PFDRT (81), PFDD (101)]	46.39 ± 10.58	28.4	n/a	n/a
Yahanda et al. (2021) [22]	PFDD• Autograft• Nonautologous	781 [Autograft (n=359), Nonautologous grafts (n=422)]	Autograft: 10.3 ± 4.6Nonautologous: 10.2 ± 4.7	Autograft: 42.3Nonautologous: 41.5	Autograft: 12;Nonautologous: 15	Autograft: 118Nonautologous: 122
Lee et al. (2014) [23]	PFD and PFDD	23 [PFDD (15), PFD (8)]	n/a	PFDD: 44PFD: 52	n/a	n/a
Jia et al. (2019) [24]	PFDD and PFDRT	115 [PFDRT (78), PFDD (37)]	PFDRT: 43.59 ± 10.44PFDD: 42.92 ± 11.66	PFDRT: 26.9PFDD: 37.8	PFDRT: 2 PFDD: 1	PFDRT: 2 PFDD: 0
Aslan et al. (2021) [25]	PFDD and PFDSD	34	n/a	n/a	n/a	n/a
Heiss et al. (2010) [26]	PFDD (reoperation)	16	35 ± 14	n/a	n/a	n/a
Jiang et al. (2018) [27]	PFDD and PFDSD	82 [PFDSD (40), PFDD (42)]	PFDSD: 13.64 (10–18)PFDD: 13.95 (10–18)	PFDSD: 50.0PFDD: 50.0	0	PFDSD: 33 PFDD: 30
Soleman et al. (2017) [28]	SSS	21 [post-FMD (16), concurrent (5)]	16.3 ± 15.4	33.3	n/a	3
Hale et al. (2020) [29]	PFD and PFDD	380 [PFD (n=74), PFDD (n=306)]	10.05 ± 4.40	40	n/a	154
Wu et al. (2012) [30]	PFDD	44	12.1 years (range 6–18)	59.1	n/a	44
Wetjen et al. (2008) [31]	PFDD	29	37 ± 12 years (range 16–61)	27.6	n/a	n/a

**Table 6:** Radiological outcomes for syrinx measurements

Author	NP	FU/months	SWPre/mm	SWPost/mm	SLPre	SLPost	S/CPre	S/CPost	VPre/cm³	Vpost/cm³	APre/mm²	APost/mm²
Han et al. (2023) [12]	FVSS (reoperation)	19.7 (2.0–70.7)	13.5	n/a	n/a	n/a	n/a	n/a	n/a	n/a	114.1 ± 81.8	24.5 ± 23.8
Gündağ Papaker et al. (2021) [13]	PFDD	58.5±22.8	5.1 ± 2.4	2.2 ± 1.0	54.9 ± 58.6 (mm)	45.6 ± 57.8 (mm)	0.84 ± 0.1	0.55 ± 0.2	n/a	n/a	n/a	n/a
Cai et al. (2023) [14]	PFDD and PFDRT	PFDD:37.8 (IQR: 30.9), PFDRT:27.2 (IQR: 31.2)	PFDD: 5.60 ± 2.01; PFDRT: 5.97 ± 2.49	PFDD: 3.73 ± 1.79; PFDRT: 2.06 ± 1.10	n/a	n/a	n/a	n/a	n/a	n/a	n/a	n/a
Geng et al. (2018) [15]	PFDS and PFDRT	PFDS: 39.24 ± 28.41; PFDRT: 43.03 ± 27.67	PFDS: 7.61 ± 3.47; PFDRT: 8.34 ± 3.45	PFDS: 5.73 ± 3.02;PFDRT: 8.09 ± 3.46	PFDS: 93.98 ± 42.70 (mm);PFDRT: 110.17 ± 43.91 (mm)	PFDS: 72.73 ± 34.79 (mm);PFDRT: 100.03 ± 44.79 (mm)	n/a	n/a	n/a	n/a	n/a	n/a
Hu et al. (2023) [16]	PFDRT or PFDD	PFDRT: 73 (32–119), PFDD:67 (31–117)	PFDRT: 5.09 ± 2.68; PFDD: 5.22 ± 2.52	PFDRT: 2.89 ± 2.15; PFDD: 2.77 ± 2.00	n/a	n/a	PFDRT: 0.56 ± 0.21; PFDD: 0.55 ± 0.21	PFDRT: 0.34 ± 0.22; PFDD: 0.36 ± 0.20	PFDRT: 4.06 ± 2.98; PFDD: 4.56 ± 4.24	PFDRT: 1.46 ± 1.85; PFDD: 1.58 ± 2.01	n/a	n/a
Akbari et al. (2022) [17]	PFDD and PFDD	PFD:32.76 ± 13.44, PFDD:32.64 ± 13.92	PFD: 6.36 ± 2.79; PFDD: 7.75 ± 3.07	PFD: 4.65 ± 2.90; PFDD: 4.36 ± 3.28	PFD: 7.44 ± 4.43;PFDD: 9.15 ± 4.61 **[segments]**	PFD: 7.02 ± 4.68;PFDD: 7.42 ± 4.29**[segments]**	n/a	n/a	n/a	n/a	n/a	n/a
Papaker et al. (2021) [18]	PFDD	80.4 ± 23.6	4.8 ± 2.6	2.4 ± 1.2	61.6 ± 71.1 (mm)	33.6 ± 51.4 (mm)	0.81 ± 0.1	0.53 ± 0.2	n/a	n/a	n/a	n/a
Wang et al. (2023) [19]	PFDD	12 (3–107)	10.27 ± 3.78	7.48 ± 4.04	n/a	n/a	n/a	n/a	n/a	n/a	n/a	n/a
Han et al. (2023) [20]	PFD, PFDD, and PFDDO	73 (IQR: 93–42.75)	n/a	n/a	n/a	n/a	n/a	n/a	Overall: 4.147 (IQR: 7.840–1.525) PFD: 4,347 (8.282.50–1.649.50) PFDD: 2,191 (7.509.50–577.05) PFDDO: 4,450 (8.046.00–2.695.88)	Overall: 1.328 (IQR: 3.627–0.553) PFD: 1,106 (5,133–638)PFDD: 916 (2,090–244)PFDDO: 1,964 (3,663–762)	n/a	n/a
Zhang et al. (2023) [21]	PFDRT and PFDD	PFDD: 23.52 ± 17.03;PFDRT: 22.23 ± 15.78	PFDRT: 5.82 ± 2.97;PFDD: 5.64 ± 3.01	n/a	n/a	n/a	PFDRT: 0.63 ± 0.22;PFDD: 0.64 ± 0.23	PFDRT: 0.35 ± 0.20;PFDD: 0.42 ± 0.22	n/a	n/a	n/a	n/a
Yahanda et al. (2021) [22]	PFDD• Autograft• Nonautologous grafts	PFDD: ≈ 31.2 ± 14.4;Autograft: ≈ 32.4 ± 14.4;Nonautologous: ≈ 30.0 ± 14.4	PFDD: 7.76 ± 3.15 • Autograft: 7.84 ± 3.13 • Nonautologous: 7.70 ± 3.16	PFDD: 4.52 ± 3.39 • Autograft: 4.31 ± 3.38• Nonautologous: 4.71 ± 3.40	PFDD: 9.0 ± 4.63• Autograft: 9.48 ± 4.80• Nonautologous: 8.65 ± 4.43 **[segments]**	PFDD: 7.36 ± 4.25 • Autograft: 7.44 ± 4.47• Nonautologous: 7.30 ± 4.02 **[segments]**	n/a	n/a	n/a	n/a	n/a	n/a
Lee et al. (2014) [23]	PFD and PFDD	PFDD: 23.5 ± 5.8;PFD: 23.7 ± 5.6	PFDD: 5.9 ± 2.5; PFD: 4.9 ± 2.4	PFDD: 2.9 mm (SD:1.7);PFD: 2.3 mm (SD:2.0) **[mean reduction reported]**	PFDD: 12.0 ± 7.0;PFD: 7.6 ± 5.3 **[segments]**	PFDD: 7.8;PFD: 4.1**[segments]**	n/a	n/a	n/a	n/a	n/a	n/a
Jia et al. (2019) [24]	PFDD and PFDRT	6 months	PFDD: 5.37 ± 2.49;PFDRT: 6.11 ± 2.81	PFDD: 3.18 ± 1.93;PFDRT: 3.48 ± 2.25	PFDD: 10.13 ± 4.42;PFDRT: 9.17 ± 4.16 **[segments]**	n/a	PFDD: 0.56 ± 0.15; PFDRT: 0.61 ± 0.19	PFDD: 0.45 ± 0.20; PFDRT: 0.46 ± 0.16	n/a	n/a	n/a	n/a
Aslan et al. (2021) [25]	PFDD and PFDSD	≥12 months minimum	PFDD: 4.24 ± 3.63PFDSD: 3.35 ± 3.80	PFDD: 2.68 ± 2.16 PFDSD: 1.68 ± 2.38	n/a	n/a	n/a	n/a	n/a	n/a	n/a	n/a
Heiss et al. (2010) [26]	PFDD (reoperation)	74.4 (8.4–158.4)	7.5 ± 3.3	2.6 ± 2.4	175 ± 135 (mm)	107 ± 118 (mm)	n/a	n/a	n/a	n/a	n/a	n/a
Jiang et al. (2018) [27]	PFDD and PFDSD	PFDSD: 35.2 (24–56)PFDD: 36.0 (range 24–48)	PFDSD: 5.2 (2–11); PFDD: 5.9 (2–15)	PFDSD: 3.4 (5.2 − 1.8) PFDD: 3.8 (5.9 − 2.1) **[Reduction= PFD= 1.8 (1–8), PFDD= 2.1 (1–12)]**	PFDSD: 9.3 (1–18)PFDD: 10.7 (3–19)**[segments]**	PFDSD: ~6.1 (9.3 − 3.2)PFDD: ~7.7 (10.7 − 3.0) **[Reduction= PFD= 3.2 (1–18), PFDD=3.0 (1–15)][segments]**	n/a	n/a	n/a	n/a	n/a	n/a
Soleman et al. (2017) [28]	SSS	Clinical: 24.9 ± 30.6;Radiologic: 26.5 ± 31.5	n/a	n/a	11.8 ± 5.4 **[segments]**	8.0 ± 6.5 **[segments]**	n/a	n/a	n/a	n/a	890 ± 463 mm²	175 ± 177 mm²
Hale et al. (2020) [29]	PFD and PFDD	118.6 ± 0.72	NSR: 8.33 ± 3.10;SR: 6.56 ± 2.89	NSR: 6.29 ± 3.09;SR: 1.54 ± 0.59	NSR: 8.85 ± 4.49;SR: 8.82 ± 4.64 **[segments]**	NSR: 8.01 ± 4.59;SR: 5.99 ± 3.89 **[segments]**	n/a	n/a	n/a	n/a	n/a	n/a
Wu et al. (2012) [30]	PFDD	45.6 (3–75.6.6)	n/a	n/a	8.1**[segments]**	3.3 **[segments]**	0.61	0.29	n/a	n/a	n/a	n/a
Wetjen et al. (2008) [31]	PFDD	36.0 ± 34.8	6.9 ± 2.1	1.1 ± 1.4	10.0 ± 5.3 **[segments]**	1.3 ± 2.6 **[segments]**	n/a	n/a	n/a	n/a	n/a	n/a

*NP* Neurosurgical Procedure, *FU* Follow-up, *SWPre* Syrinx width pre-operation, *SWPost* Syrinx width post-operation, *SLPre* Syrinx length pre-operation, *SLPost* Syrinx length post-operation, *S/CPre* Syrinx-to-cord ratio pre-operation, *S/CPost* Syrinx-to-cord ratio post-operation, *VPre* Volume of syrinx pre-operation, *VPost* Volume of syrinx post-operation, *APre* Area of syrinx pre-operation, *APost* Area of syrinx post-operation, *NSR* No syrinx resolution, *SR* Syrinx resolution

**Table 7:** Summary of reported stable, worsened, improved, or resolved syrinxes

Author	Unchanged syrinx size/number	Complete syrinx resolution/number	Syrinx reduction (but incomplete resolution)/number	Worsened syrinx/number
Han et al. (2023) [12]	0	0	13	0
Gündağ Papaker et al. (2021) [13]	1	3	4	0
Cai et al. (2023) [14]	n/a	n/a	n/a	n/a
Geng et al. (2018) [15]	PFDS: 6; PFDRT:7	n/a	PFDS: 8; PFDRT: 24	PFDS:3;PFDRT:1
Hu et al. (2023) [16]	n/a	n/a	n/a	n/a
Akbari et al. (2022) [17]	n/a	n/a	PFDD: 216;PFD: 32	n/a
Papaker et al. (2021) [18]	3	12	8	n/a
Wang et al. (2023) [19]	8	22	220	15
Han et al. (2023) [20]	n/a	n/a	PFD: 22;PFDD: 20;PFDDO: 36	0
Zhang et al. (2023) [21]	n/a	PFDRT: 8;PFDD: 5	n/a	PFDRT: 12; PFDD: 17[**same/worse**]
Yahanda et al. (2021) [22]	n/a	n/a	n/a	n/a
Lee et al. (2014) [23]	n/a	n/a	PFDD: 10 PFD: 8 **[improved/resolved] **	n/a
Jia et al. (2019) [24]	PFDRT: 22 PFDD: 7	n/a	PFDRT: 54 PFDD: 29	PFDRT: 2 PFDD: 1
Aslan et al. (2021) [25]	n/a	n/a	n/a	n/a
Heiss et al. (2010) [26]	1	n/a	15	0
Jiang et al. (2018) [27]	n/a	n/a	PFDSD: 33PFDD: 38**[improved/resolved]**	n/a
Soleman et al. (2017) [28]	1	n/a	20	0
Hale et al. (2020) [29]	235	n/a	145	44
Wu et al. (2012) [30]	1	20	23	0
Wetjen et al. (2008) [31]	n/a	n/a	3–6 months: 25/29;1 yr: 21/23;2yr: 19/19*	0

* Value used for analysis, as our study included the most recent follow-up values

### Meta-analyses

#### Radiological changes following PFDD

A. Mean change in syrinx width for patients undergoing PFDD

**Fig. 2:**
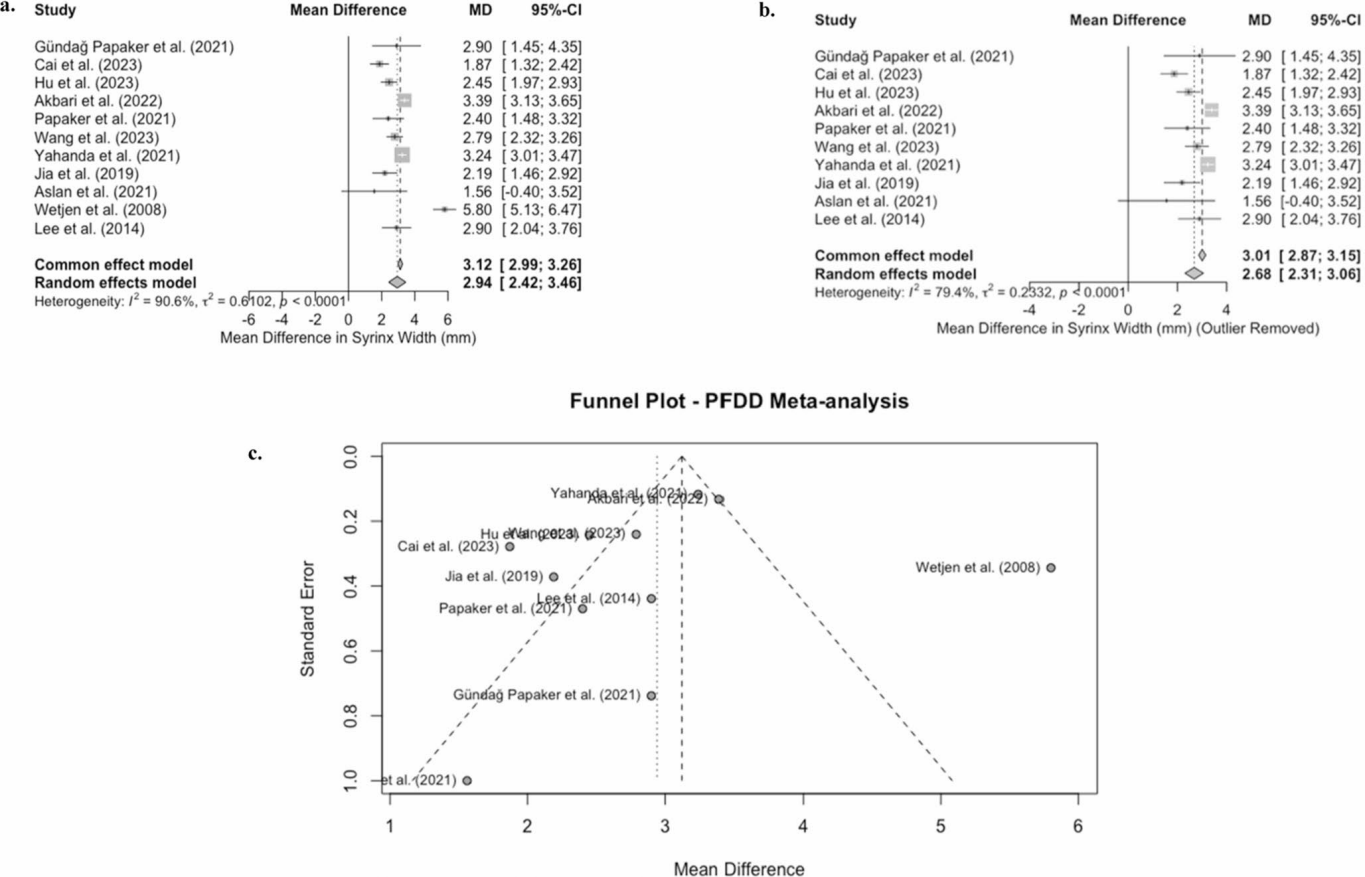
Forest & funnel plots for mean change in syrinx width following PFDD

A meta-analysis using a random-effects model was conducted on 10 studies evaluating changes in syrinx width after surgery. Our results showed a significant reduction in mean syrinx width following surgery (*p* < 0.0001). The pooled mean difference (MD) was 2.94 mm (95% CI: 2.42–3.46) (Fig. 2a). However, there was significant heterogeneity (I2 = 90.6%, t2 = 0.6102, *p* < 0.0001), indicating substantial variability between studies.

A funnel plot (Fig. 2c) was used to assess potential publication bias, and upon visual inspection, mild asymmetry was noted. Wetjen et al. [31] were identified as a potential outlier, exhibiting a significantly larger effect size. A formal test for funnel plot asymmetry was also conducted using Egger’s linear regression, and the test did not suggest significant publication bias. The results of the test were t= −0.72, degrees of freedom = 9, and p-value = 0.4880. The interception bias estimate was − 1.3806 (SE: 1.9093), and the residual heterogeneity variance was high t2, thus supporting the use of the random-effects model.

We conducted a sensitivity analysis by excluding the outlier by Wetjen et al. [31], which had a considerable effect size (MD: 5.80 mm). Following this exclusion, the heterogeneity of the analysis decreased to I2 = 79.4%, t2 = 0.2332, *p* < 0.0001, and the mean difference (MD) was 2.68 mm (95% CI: 2.31–3.06) (*p* < 0.0001) (Fig. 2b).

The results have shown a significant reduction in syrinx width following the PFDD procedure, but high-influence outliers moderately influence the magnitude of this reduction. There was no evidence of publication bias detected; however, significant heterogeneity remained even after excluding the notable outlier.

B. Mean change in Syrinx length (vertebral segments) in PFDD

**Fig. 3:**
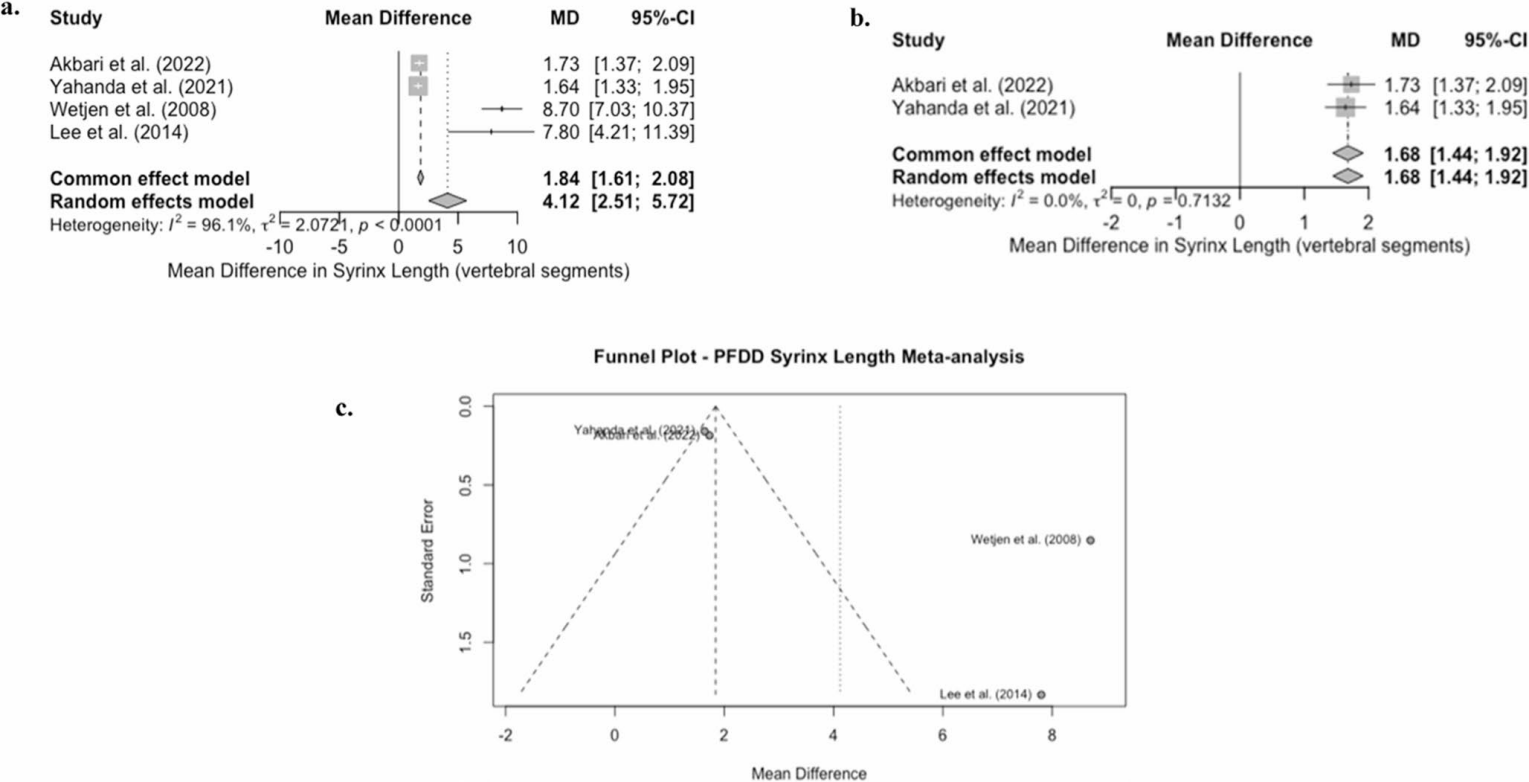
Forest & funnel plots for mean change in syrinx length following PFDD

A random-effects meta-analysis was done on an initial set of four studies reporting syrinx length in vertebral segments. The pooled mean difference (MD) for this analysis was 4.12 (95% CI, 2.51 to 5.72) (*p* < 0.0001), indicating a significant reduction in syrinx length (Fig. 3a). However, the heterogeneity of this analysis was very high, with (I2 = 96.1%, t2 = 2.0721, *p* < 0.0001), which suggests significant differences between study results. Lee et al. [23] and Wetjen et al. [31] were notable outliers, as they had substantial effect sizes (MD of 7.8 and 8.7, respectively).

To assess publication bias, we visually inspected the funnel plot generated (Fig. 3c), which shows apparent asymmetry. The two outliers mentioned previously fell far outside the boundaries of the funnel plot, which raises concerns about potential publication bias or methodological differences.

We conducted a sensitivity analysis, excluding these outliers, and retained the two homogeneous studies: Akbari et al. [17] and Yahanda et al. [22]. The updated analysis yielded a pooled mean difference of 1.68 vertebral segments (95% CI: 1.44–1.92) (*p* < 0.0001) under a random-effects model (Fig. 3b).

These findings suggest that PFDD leads to a significant reduction in syrinx length; however, our analysis was significantly influenced by outliers in the study. A more conservative pooled result from our sensitivity analysis is a better representation of the typical clinical impact. In contrast, the result from the initial analysis is likely to be overestimated due to high-variance studies with extreme outcomes.

 C. Mean change in S/C ratio for patients undergoing PFDD

**Fig. 4:**
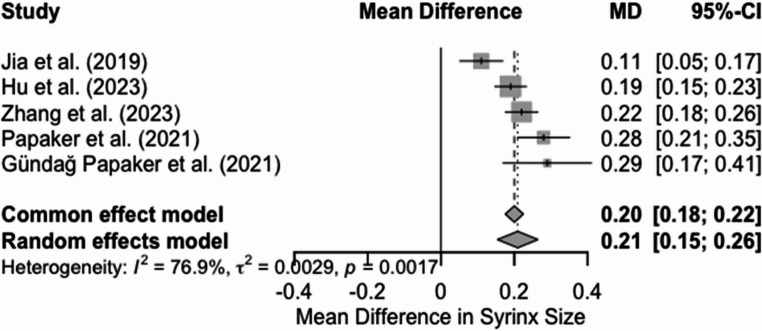
Forest plot for mean change in S/C ratio following PFDD

Four studies were included in the random-effects meta-analysis on mean change in S/C ratio following PFDD. The pooled mean change following this analysis was 0.21(*p* < 0.0001), with confidence intervals of 0.15 to 0.26. However, there was notable heterogeneity (I2 = 76.9%, t2 = 0.0029, *p* = 0.0017) (Fig. 4), suggesting variability across studies in the magnitude of effect. The funnel plot generated (Sf 1) showed a fairly symmetrical distribution upon visual inspection around the pooled effect size, with no clear suggestion of publication bias or minor study effects.

D. Proportion of Syrinx resolution or improvement in patients undergoing PFDD

**Fig. 5:**
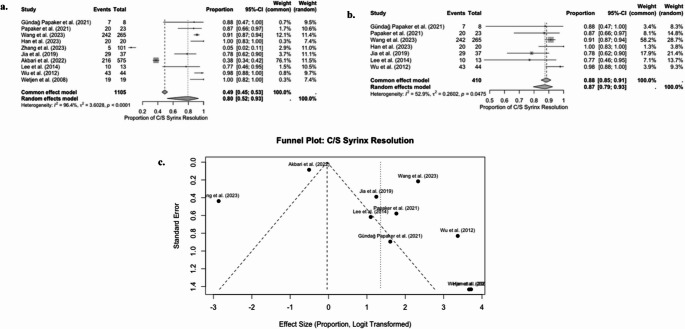
Forest & funnel plots for proportion of syrinx resolution/improvement following PFDD

A meta-analysis of 10 studies was conducted to analyse the proportion of patients with syrinx resolution or improvement in syrinx size following PFDD. The random-effects model analysis yielded a pooled estimate of 0.80 (95% CI, 0.52–0.93) (*p* < 0.05) (Fig. 5a). The heterogeneity of the analysis was very high (I2 = 96.4%, τ2 = 3.6028, *p* < 0.0001), suggesting variance between studies.

A funnel plot of logit-transformed effect sizes was generated (Fig. 5c), revealing visual asymmetry. Studies such as Wetjen et al. [31] and Wu et al. [30] reported unusually high effect sizes with low standard errors. In contrast, Akbari et al. [17] and Zhang et al. [21] had lower proportions and larger variances.

An Egger’s test was then conducted to test for asymmetry, and the results indicated no statistically significant evidence of publication bias (t = 1.62, df = 8, p-value = 0.1443). The bias estimate was reported as 3.3352 (SE = 2.0613). Notably, the residual heterogeneity of the test remained high (t2 = 23.51), thereby limiting the reliability of the asymmetry test.

We conducted a sensitivity analysis by excluding Akbari et al. [17], Zhang et al. [21], and Wetjen et al. [31], which were outliers contributing to the funnel plot asymmetry. The restricted analysis included seven studies, and the pooled random effects proportions were 0.87 (95% CI: 0.79 to 0.93) (*p* < 0.05) (Fig. 5b). The heterogeneity of the studies was reduced to a more moderate level (I2 = 52.9%, τ2 = 0.2602, *p* = 0.0475).

PFDD appears to be associated with a high likelihood of syrinx resolution, with pooled estimates ranging from 0.80 to 0.88. Despite this, there is substantial heterogeneity, funnel plot asymmetry, and variation in study quality, which necessitates careful interpretation of the findings.

 E. Proportion of worsened or unchanged syrinx size following PFDD

**Fig. 6:**
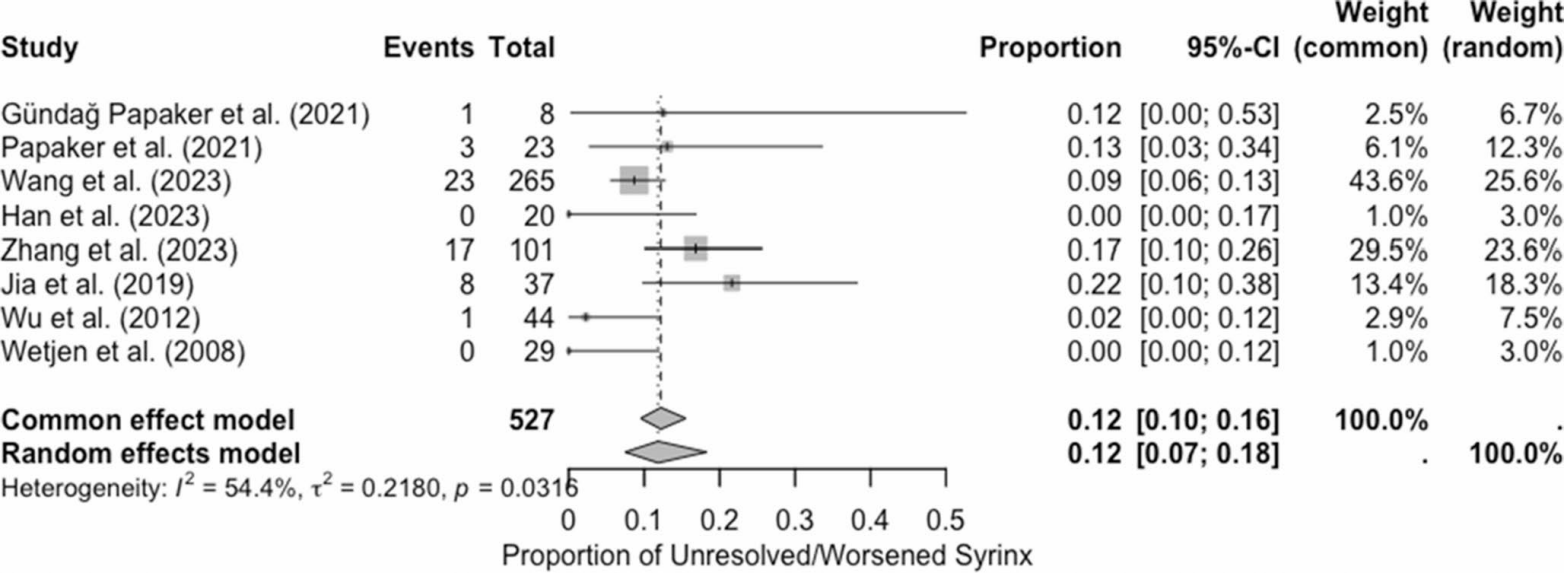
Forest plot for worsened/unchanged syrinx following PFDD

A random-effects meta-analysis on seven studies assessing the proportion of patients with unchanged or worsened syrinx following PFDD generated a pooled proportion of 0.12 with a 95% CI of 0.07 to 0.18 (*p* < 0.05). There was moderate heterogeneity for the analysis (I2 = 54.4%, t2 = 0.2180, *p* = 0.0316) (Fig. 6). This suggests that a minority of patients had experienced worsening or unchanged syrinx following treatment with PFDD, with only 12% demonstrating no improvement or worsening syrinx. Mild visual asymmetry was noted upon visual inspection of the funnel plot generated (Sf 2); however, the plot remained largely symmetrical around the pooled estimate.

#### Radiological changes following PFDRT

A. Mean change in syrinx width for patients undergoing PFDRT

**Fig. 7:**
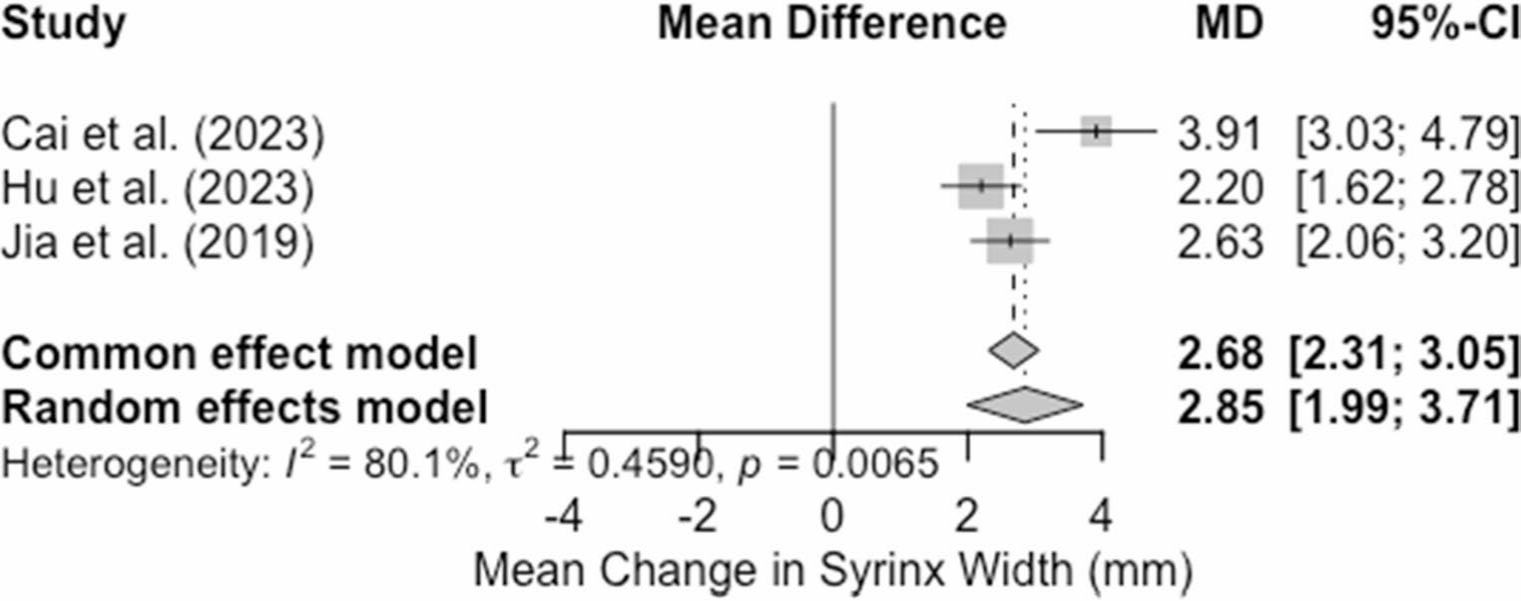
Forest plot for mean change in syrinx width following PFDRT

Three studies were included in the meta-analysis, which used a random-effects model to calculate the mean change in syrinx width for patients undergoing PFDRT. The meta-analysis revealed a significant reduction in syrinx width, with a pooled mean difference (MD) of 2.85 mm (95% CI: 1.99–3.71) (*p* < 0.0001) (Fig. 7). Similarly, the analysis also yielded high heterogeneity (I2 = 80.1%, τ2 = 0.4590, *p* = 0.0065), indicating significant variability between the studies. Visual inspection of the funnel plot revealed mild asymmetry, with Cai et al. [14] potentially influencing the overall pooled estimate, as it contributes to a larger effect size and has a minor standard error (Sf 3).

#### Comparisons between PFDRT vs. PFDD

A) Mean change in syrinx width for patients undergoing PFDRT vs. PFDD

**Fig. 8:**
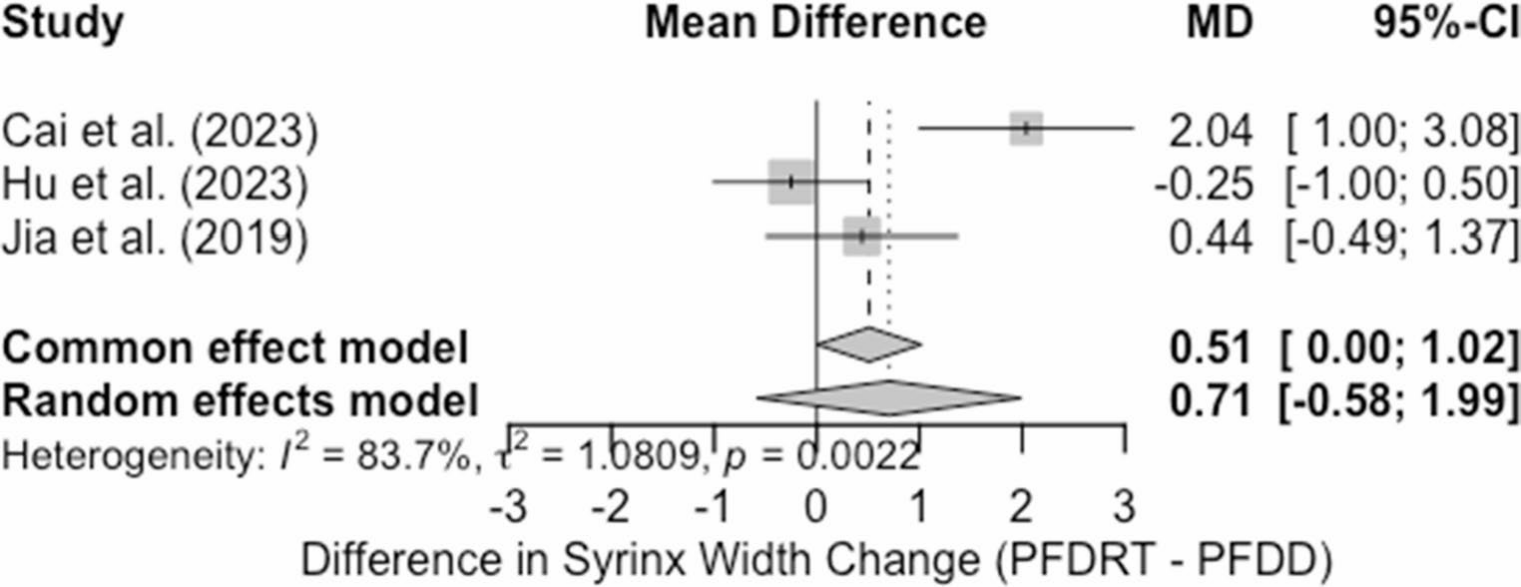
Forest plot comparing the mean change in syrinx width between PFDRT and PFDD

Three studies comparing PFDD with PFDRT were included in the random-effects meta-analysis for reduction of syrinx width. This yielded a non-significant mean difference of 0.71 mm (95% CI: −0.58 to 1.99, *p* = 0.283) (Fig. 8). The heterogeneity was also high for the study (I2 = 83.7%, t2 = 1.0809, *p* = 0.0022). Therefore, suggesting significant variability between study results. The direction of the effect favoured PFDRT. However, the confidence interval crossed the null. A funnel plot has shown visual asymmetry (Sf 4), but this may reflect random variation rather than actual bias, given the minimal number of studies included in the analysis.

B) Mean change in S/C ratio for patients undergoing PFDRT vs. PFDD

**Fig. 9:**
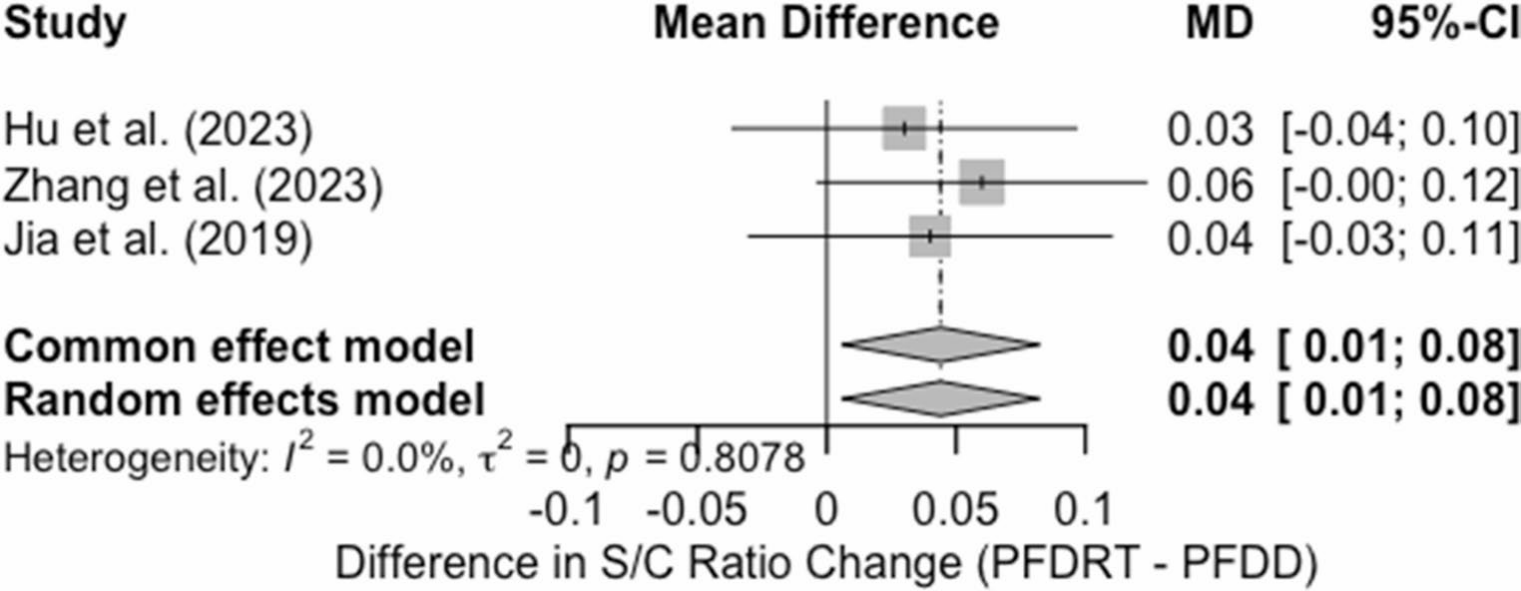
Forest plot comparing the mean change in S/C ratio between PFDRT and PFDD

Studies by Hu et al. [16], Zhang et al. [21], and Jia et al. [24] were included for a meta-analysis comparing the changes in S/C ratio between PFDD and PFDRT neurosurgical procedures. The results showed a pooled mean difference (MD) of 0.04 with a 95% confidence interval (CI) of 0.01 to 0.08, favouring PFDRT (*p* = 0.0248), which was statistically significant. The analysis also revealed no heterogeneity (I2 = 0.0%, τ2 = 0, *p* = 0.8078) (Fig. 9), indicating consistent findings. Despite this, the slight magnitude difference might have limited clinical significance. The funnel plot generated showed visual symmetry (Sf 5), consistent with a low likelihood of reporting bias. The number of studies included was minimal, making the results less reliable.

C) CCOS scores for PFDRT vs. PFDD

**Fig. 10:**
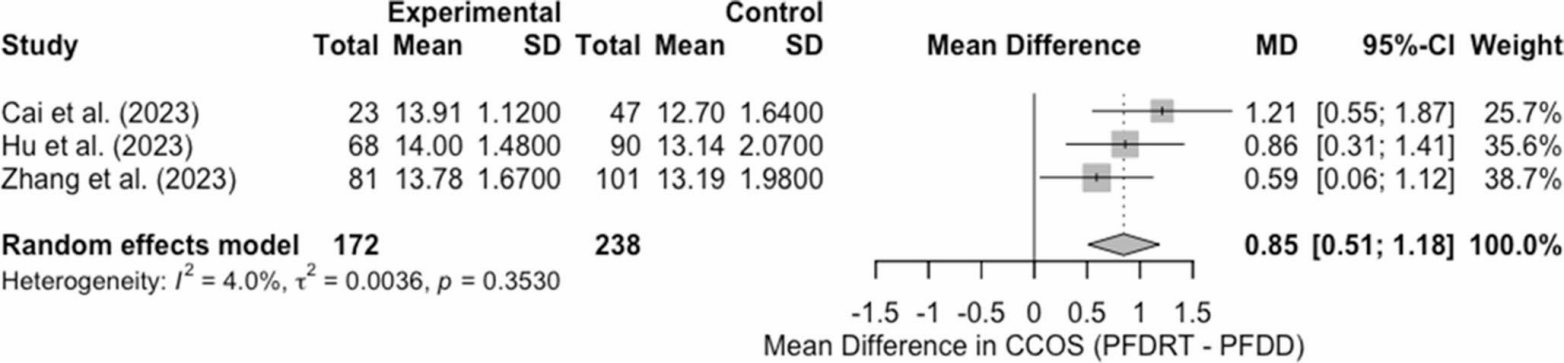
Forest plot comparing CCOS scores between PFDRT and PFDD

A random-effects meta-analysis was conducted to evaluate differences in CCOS scores between PFDD and PFDRT. This included 3 of the 20 studies included in our final analysis. he pooled analysis revealed a statistically significant improvement in CCOS scores among patients in the PFDRT group compared to those in the PFDD group. The pooled mean difference was 0.85 (95% CI: 0.51 to 1.18) (*p* < 0.0001) (Fig. 10), indicating a moderate and consistent benefit favouring PFDRT over PFDD. The study heterogeneity was also low, with an I2 value of 4.0%, t2 = 0.0036, and a Cochran’s Q test p-value of 0.3530, further supporting the consistency of the findings. A visual assessment of the funnel plot showed no significant asymmetry (Sf 6).

## Discussion

### Key findings

Our systematic review and meta-analysis included 20 studies, comprising both randomised and non-randomised studies, with over 3,000 patients. Through our study, we found that PFDD and PFDRT had statistically significant effects in reducing various metrics for syrinx size. The radiological improvements seen were most consistent in measures such as syrinx width and S/C ratio. PFDD has been shown to provide meaningful reductions in syrinx width (MD: 2.68 mm), syrinx length (MD: 1.68 segments), and S/C ratio (MD: 0.21). PFDD was also associated with a high proportion of qualitative syrinx improvement (0.88) and a low proportion of unchanged or worsened syrinx (MD: 0.12). Our head-to-head analysis has indicated that PFDRT was more effective in reducing syrinx size, as measured by syrinx width and S/C ratios. However, only the analysis of the S/C ratio yielded statistically significant results. The analysis of CCOS scores between the two procedures also favoured PFDRT by 0.85.

### The context within the literature

Studies by Gallo et al. [4, 32] and Osborne-Grinter et al. [33] have highlighted the benefits of PFDD compared to other decompression techniques in the context of CM1 patients. In their study, PFDD was reported to have been associated with improved clinical outcomes, shorter hospital stays, and lower complication risks within the adult population of CM1 [4]. Lower complication rates were also seen within the paediatric population with CM1, where PFDD was performed [32]. Our study supports the current evidence by also suggesting that the use of PFDD is associated with positive radiological outcomes in patients with CM1 + syrinx. Even though our head-to-head analysis between PFDD and PFDRT favoured PFDRT (in S/C ratio and CCOS), the magnitude of the benefit was relatively small. While there is evidence suggesting that reductions in syrinx-to-cord ratio are positively correlated with CCOS [34], several other studies emphasise that syrinx resolution is not a sole determinant for better prognosis amongst adult and pediatric patients and usually involves a multitude of factors such as the type and duration of symptoms [35–37]. Importantly, the lack of evidence makes it difficult to determine at what magnitude of S/C ratio reduction is considered beneficial. Therefore, determining whether PFDRT would be superior to PFDD would also largely depend on several other factors, such as complication risks and the length of the procedure. Notably, further comparative studies are also needed to determine if PFDRT would be consistent in reducing other syrinx metrics such as width, length and volume.

As mentioned, syrinx resolution does not necessarily equate to clinical recovery, and there is existing controversy within the literature regarding the use of imaging alone to determine the efficacy of neurosurgical techniques. While several scoring systems exist, such as CCOS [38], these have limitations, and emerging scoring systems, like SOSXW, lack validation [39]. There is a lack of a gold standard clinical scoring system, and a tool combining both imaging and clinical outcomes would best inform surgical practice.

Additionally, it is also essential to interpret surgical outcomes alongside the natural history of CM1 + syringomyelia. Longitudinal data in a recent study by Yuan et al. [40] has shown that spontaneous resolution of CM1 + syringomyelia was more common in cervical syringes and in patients without obstructive sleep apnoea-hypopnoea syndrome, and highlights the variable progressions seen within the CM1 + syringomyelia population. Including natural history comparators would further enhance the validity of individual studies and delineate actual treatment effects from underlying disease variability.

### Other techniques

Beyond posterior fossa decompression: our review has also identified two studies reporting the use of FVS and SSS as salvage techniques in patients with persistent or recurrent syringomyelia post-decompression surgery. These techniques demonstrated high success rates, resulting in improved or resolved syrinxes in 100% and 95% of their patients, respectively. The FVS helps provide a channel, enabling CSF flow from the fourth ventricle into the subarachnoid space, thereby bypassing any obstructions. SSS works similarly, utilising a flexible tube to redirect fluid within the syrinx to the subarachnoid space, thereby relieving pressure [41]. While our review did not include a formal meta-analysis due to the limited data available in the literature, recent case series and small cohort studies have supported the use of these techniques in specific patient subgroups, including those with large syrinxes [42–44] and children presenting with hydrocephalus [6]. Further prospective trials would be encouraged to help establish long-term efficacy and enable comparisons with conventional decompression techniques.

### Strengths

This study fills a significant gap in the literature by providing a quantitative analysis of syrinx reduction through radiological measurements. Studies such as Antkowiak & Tabakow et al. [45] provide meaningful insights into complication risks, CSF leaks, and neurologic deficits for patients with CM1 and syringomyelia, as well as the neurosurgical techniques mentioned in this study (i.e., PFD, PFDD, and PFDRT). However, when assessing syringomyelia resolution, this was only reported qualitatively. Similarly, Perrini et al. [46] also provide limited quantification of syrinx metrics, focusing on outcomes such as the rate of postoperative syrinx shrinkage. Therefore, this study represents the first systematic review and meta-analysis to quantitatively evaluate radiological outcomes across multiple neurosurgical techniques for treating CM1 + syringomyelia.

### Limitations

Our study has several limitations. Some of the studies included in our meta-analysis had a relatively small sample size, which could have affected the statistical power of our subgroup comparisons. Studies included in our analysis consisted of observational studies, and no RCTs were incorporated. Furthermore, due to the limited number of studies included in our head-to-head analyses (three in each), the results should be interpreted as hypothesis-generating rather than conclusive.

Additionally, there was also considerable heterogeneity across our analysis, likely attributed to several factors. First, our analysis pooled both pediatric and adult cases due to the limited number of available studies reporting pre- and postoperative radiological measures for each surgical technique. Moreover, important demographic variables such as sex distribution, age, preoperative symptoms, race, and ethnicity were inconsistently reported, particularly by studies that included CM1 patients with and without syringomyelia. The lack of stratified demographic data could have further influenced heterogeneity in our analysis [47]. Some studies may also have slightly different variations of conducting a specific surgical technique. For example, materials used for duraplasty in PFDD might vary between studies, with some using autograft and others using non-autologous materials. Lastly, definitions for syrinx resolution were not standardised across studies, and individual studies used different thresholds to define resolution or improvement, which contributed to the high heterogeneity observed in our subgroup analysis for improved or resolved syrinx.

Another significant limitation was that our study did not report reoperation rates, as most of our included studies did not consistently report this variable for the CM1 + syrinx population. Reoperation is a clinically important outcome that may reflect insufficient decompression, disease progression or overall effectiveness of the surgical procedure. In a similar systematic review focused on treatments for patients with syringomyelia (without CM1), incidences of revision surgery were one of the key outcomes utilised to determine comparative efficacy between syringosubarachnoid, syringoperitoneal, and syringopleural shunting [48]. Notably, a large-scale cohort study (*n* = 528) on CM1 has highlighted that revision surgeries were significantly more frequent in patients undergoing bony and dural decompression compared to patients who had undergone anatomical reduction of herniated tonsils and cerebrospinal fluid decompression [49]. This emphasises the need for future studies to standardise reporting of reintervention data, as it ultimately informs better surgical decision-making and better patient outcomes.

### Future recommendations

Future studies should focus on standardising radiological outcome reporting and incorporating more prospective designs, including randomised controlled trials, to strengthen the evidence base. To our knowledge, a recent trial comparing PFD with PFDD has already been completed [50], and other trials comparing PFDD with PFDRT in Beijing [51] and the UK Chiari 1 study [52] are currently underway. RCTs in regions such as India and China, where craniovertebral junction abnormalities are more prevalent, may also be warranted to understand race or sex differences in affecting syringomyelia resolution. Results from these studies would enable more robust meta-analyses to be conducted; however, conducting such trials poses a challenge, as they require larger sample sizes and funding.

In addition to imaging outcomes, future directions should also focus on CSF dynamics. Key foundational studies by Stoodley et al. [53] and Heiss et al. [54] have provided a meaningful understanding of the pathophysiology of syringomyelia formation. Utilising these pathophysiological insights would help improve patient outcomes. Therefore, studies on the craniocervical junction and spinal malformations, as well as their effects on abnormal CSF circulation mediated by mechanisms such as cilia and scoliosis [55, 56], may inform novel surgical techniques [57] that could better manage syringomyelia in CM1 patients. In support of this, recent mechanistic insights by Yuan et al. [58] have demonstrated the restoration of CSF flow through the cranial end of the central canal in patients undergoing foramen magnum and foramen of Magendie dredging for post-traumatic syringomyelia. Understanding how CSF redistributes following decompression has provided a meaningful physiological framework that may help reinforce the rationales behind existing decompressive techniques for syringomyelia in CM1 patients.

## Conclusion

This systematic review and meta-analysis quantitatively evaluated outcomes across multiple neurosurgical techniques for CM1 + syringomyelia. The results from our study have shown that PFDD and PFDRT are associated with consistent reductions in syrinx dimensions, including width, length, and S/C ratio. Furthermore, PFDRT demonstrated a modest but statistically significant advantage over PFDD in reducing S/C ratio and improving CCOS scores. While other studies primarily relied on subjective or binary outcomes, our review has provided objective, quantifiable radiological improvement stratified by neurosurgical techniques. The results from our study provide preliminary evidence that helps inform surgical decision-making, particularly when determining which surgical technique is most likely to yield an optimal radiological response. However, due to the limited effect sizes and the lack of directly comparative studies, our results should be interpreted with caution. Future studies should correlate radiological syrinx metric changes with clinical outcomes more effectively to help establish the long-term effectiveness of these interventions. Additionally, further research should explore underutilised neurosurgical techniques.

## Supplementary Information

Below is the link to the electronic supplementary material.

Supplementary Material 1 (PDF 134 KB)

Supplementary Material 2 (DOCX 62.8 KB)

Supplementary Material 3 (DOCX 65 KB)

Supplementary Material 4 (DOCX 55.8 KB)

Supplementary Material 5 (DOCX 56.9 KB)

Supplementary Material 6 (DOCX 58.8 KB)

Supplementary Material 7 (DOCX 54 KB)

## Data Availability

No datasets were generated or analysed during the current study.

## References

[1] Kular S, Cascella M, Chiari I (2020) Malformation [Internet]. PubMed. Treasure Island (FL): StatPearls Publishing; Available from: https://www.ncbi.nlm.nih.gov/books/NBK554609/

[2] Arnautovic A, Splavski B, Boop FA, Arnautovic KI (2015) Pediatric and adult Chiari malformation type I surgical series 1965–2013: a review of demographics, operative treatment, and outcomes. Journal of Neurosurgery: Pediatrics 15(2):161–177 25479580 10.3171/2014.10.PEDS14295

[3] Tam SKPo, Brodbelt A, Bolognese PA, Foroughi M (2020) Posterior fossa decompression with duraplasty in Chiari malformation type 1: a systematic review and meta-analysis. Acta Neurochir 163(1):229–238 32577895 10.1007/s00701-020-04403-9

[4] Gallo P, Copley PC, McAllister S, Kaliaperumal C (2021) The impact of neurosurgical technique on the short- and long-term outcomes of adult patients with Chiari I malformation. Clin Neurol Neurosurg 200:106380 33387726 10.1016/j.clineuro.2020.106380

[5] Zhang L, Li BL, Wei S, Hu HW, Chen HF, Fan YC et al (2025) Clinical efficacy of surgery for patients with Chiari malformation type I with syringomyelia: posterior fossa decompression versus posterior fossa decompression with resection of tonsils. Front Neurol 16:1556026 10.3389/fneur.2025.1556026 40098683 10.3389/fneur.2025.1556026 PMC11912941

[6] Sun P, Zhou M, Liu Y, Du J, Zeng G (2022) Fourth ventricle stent placement for treatment of type I Chiari malformation in children. Childs Nerv Syst 39(3):671–676 36572815 10.1007/s00381-022-05793-0

[7] Aydin L, Dereli D, Kartum TA, Sirinoglu D, Sahin B, Eksi MS et al (2024) Management of persistent syringomyelia in patients operated for Chiari malformation type 1. World Neurosurg 182:e360–e368 38013110 10.1016/j.wneu.2023.11.109

[8] Wells G, Shea B, O’Connell D, Peterson J, Welch V, Losos M et al The Newcastle-Ottawa Scale (NOS) for assessing the quality of nonrandomised studies in meta-analyses [Internet]. www.ohri.ca.2021. Available from: https://www.ohri.ca/programs/clinical_epidemiology/oxford.asp

[9] Munn Z, Barker TH, Moola S, Tufanaru C, Stern C, McArthur A et al (2020) Methodological Quality of Case Series Studies. JBI Database of Systematic Reviews and Implementation Reports [Internet]. ;18(10):1. Available from: https://pubmed.ncbi.nlm.nih.gov/33038125/

[10] Cochrane (2019) RoB 2: A revised Cochrane risk-of-bias tool for randomized trials [Internet]. Cochrane.org. Available from: https://methods.cochrane.org/bias/resources/rob-2-revised-cochrane-risk-bias-tool-randomized-trials

[11] Chapter 6 Choosing effect measures and computing estimates of effect | Cochrane [Internet]. Cochrane.org. 2023 [cited 2025 Jun 25]. Available from: https://www.cochrane.org/authors/handbooks-and-manuals/handbook/current/chapter-06

[12] Han RK, Medina MP, Giantini-Larsen AM, Chae JK, Cruz A, Garton ALA et al (2023) Fourth ventricular subarachnoid stent for Chiari malformation type I–associated persistent syringomyelia. Neurosurg Focus 54(3):E10 36857783 10.3171/2022.12.FOCUS22633

[13] Gündağ Papaker M, Abdallah A, Çınar İ (2021) Surgical Outcomes of Adult Chiari Malformation Type 1: Experience at a Tertiary Institute. Cureus 10.7759/cureus.17876 PMC8502452 34660075

[14] Cai Y, Wang C, Chai S, Li G, Zhang T, Liu Z et al (2023) Prognostic analysis of posterior fossa decompression with or without cerebellar tonsillectomy for Chiari malformation type I: a multicenter retrospective study. NeuroSurg Focus 54(3):E4 36857790 10.3171/2022.12.FOCUS22626

[15] Geng LY, Liu X, Zhang YS, He SX, Huang QJ, Liu Y et al (2018) Dura-splitting versus a combined technique for Chiari malformation type I complicated with syringomyelia. Br J Neurosurg 32(5):479–483 30146911 10.1080/02688697.2018.1498448

[16] Hu Y, Zhang M, Duan C, Song D, Wei M, Guo F (2023) A long-term follow-up study of adults with Chiari malformation type I combined with syringomyelia. Front Neurol 14:1274971 10.3389/fneur.2023.1274971 38107634 10.3389/fneur.2023.1274971 PMC10722987

[17] Akbari HA, Yahanda AT, Ackerman LL, Adelson PD, Ahmed R, Albert GW et al (2022) Complications and outcomes of posterior fossa decompression with duraplasty versus without duraplasty for pediatric patients with Chiari malformation type I and syringomyelia: a study from the Park-Reeves Syringomyelia research consortium. J Neurosurg Pediatr 30(1):39–51 35426814 10.3171/2022.2.PEDS21446

[18] Papaker MG, Abdallah A, Cesme DH, Gönen G, Asiltürk M, Avyasov R et al (2020) Clinical and radiological evaluation of treated Chiari I adult patients: retrospective study from two neurosurgical centers. Neurosurg Rev 44(4):2261–2276 33051726 10.1007/s10143-020-01414-z

[19] Wang B, Wang C, Zhang YW, Liang YC, Liu WH, Yang J et al Long-term outcomes of foramen magnum decompression with duraplasty for Chiari malformation type I in adults: a series of 297 patients. Neurosurgical FOCUS [Internet]. 2023 Mar 1 [cited 2025 Mar 18];54(3):E5–5. Available from: https://thejns.org/focus/view/journals/neurosurg-focus/54/3/article-pE5.xml 10.3171/2022.12.FOCUS22627 36857791

[20] Han S, Hou B, Li Z, Feng F, Li Y, Gao J (2023) Individualized functional decompression options for adult Chiari malformation with Syringomyelia and a novel scale for Syringomyelia resolution: a single-center experience. Neurospine 20(4):1501–1512 38171316 10.14245/ns.2346626.313 PMC10762401

[21] Zhang M, Hu Y, Song D, Duan C, Wei M, Zhang L et al (2023) Exploring the prognostic differences in patients of Chiari malformation type I with Syringomyelia undergoing different surgical methods. Front Neurol 13:1062239 10.3389/fneur.2022.1062239 36686516 10.3389/fneur.2022.1062239 PMC9846178

[22] Yahanda AT, Adelson PD, Akbari HA, Albert GW, Aldana PR, Alden TD et al (2021) Dural augmentation approaches and complication rates after posterior fossa decompression for Chiari I malformation and syringomyelia: a Park-Reeves Syringomyelia research consortium study. J Neurosurg Pediatr 27(4):459–468 33578390 10.3171/2020.8.PEDS2087

[23] Lee A, Yarbrough CK, Greenberg JK, Barber J, Limbrick DD, Smyth MD (2014) Comparison of posterior fossa decompression with or without duraplasty in children with type I Chiari malformation. Childs Nerv Syst 30(8):1419–1424 24777296 10.1007/s00381-014-2424-5 PMC4104143

[24] Jia C, Li H, Wu J, Gao K, Cheng B, Zhao, Li M et al (2019) Comparison decompression by duraplasty or cerebellar tonsillectomy for Chiari malformation-I complicated with Syringomyelia. Clinical neurology and neurosurgery 176:1–7 10.1016/j.clineuro.2018.11.008 30448544

[25] Aslan A, Rakip U, Boyacı MG, Yildizhan S, Kormaz S, Atay E et al (2020) Posterior fossa decompression and superficial durotomy rather than complete durotomy and duraplasty in the management of Chiari 1. Neurol Res 43(6):440–446 33357109 10.1080/01616412.2020.1866386

[26] Heiss JD, Suffredini G, Smith R, DeVroom HL, Patronas NJ, Butman JA et al (2010) Pathophysiology of persistent syringomyelia after decompressive craniocervical surgery. Journal of neurosurgery. Spine 13(6):729–742 21121751 10.3171/2010.6.SPINE10200 PMC3822767

[27] Jiang E, Sha S, Yuan X, Zhu W, Jiang J, Ni H et al (2018) Comparison of clinical and radiographic outcomes for posterior fossa decompression with and without duraplasty for treatment of pediatric Chiari I malformation: a prospective study. World Neurosurg 110:e465–e472 29133007 10.1016/j.wneu.2017.11.007

[28] Soleman J, Roth J, Bartoli A, Rosenthal D, Korn A, Constantini S (2017) Syringo-subarachnoid shunt for the treatment of persistent syringomyelia following decompression for Chiari type I malformation: surgical results. World Neurosurg 108:836–843 28807779 10.1016/j.wneu.2017.08.002

[29] Hale AT, Adelson PD, Albert GW, Aldana PR, Alden TD, Anderson RCE et al (2020) Factors associated with syrinx size in pediatric patients treated for Chiari malformation type I and syringomyelia: a study from the Park-Reeves syringomyelia research consortium. Journal of Neurosurgery: Pediatrics 25(6):629–639 32114543 10.3171/2020.1.PEDS19493

[30] Wu T, Zhu Z, Jiang J, Zheng X, Sun X, Qian B et al (2011) Syrinx resolution after posterior fossa decompression in patients with scoliosis secondary to Chiari malformation type I. Eur Spine J 21(6):1143–1150 22086538 10.1007/s00586-011-2064-3 PMC3366146

[31] Wetjen NM, Heiss JD, Oldfield EH (2008) Time course of Syringomyelia resolution following decompression of Chiari malformation type I. Journal of Neurosurgery: Pediatrics 1(2):118–123 18352779 10.3171/PED/2008/1/2/118 PMC4294217

[32] Gallo P, Sokol D, Chandrasekaran Kaliaperumal, Kandasamy J (2017) Comparison of three different Cranio-Cervical decompression procedures in children with Chiari malformation type I: does the surgical technique matter? Pediatr NeuroSurg 52(5):289–297 28848212 10.1159/000479327

[33] Osborne-Grinter M, Arora M, Kaliaperumal C, Gallo P (2021) Posterior fossa decompression and duraplasty with and without arachnoid preservation for the treatment of adult Chiari malformation type 1: a systematic review and meta-analysis. World Neurosurg 151:e579–e598 33940274 10.1016/j.wneu.2021.04.082

[34] Lu C, Ma L, Guan J, Liu Z, Wang K, Duan W et al (2022) Relationship between syrinx resolution and cervical sagittal realignment following decompression surgery for Chiari I malformation related syringomyelia based on configuration phenotypes. Neurospine 19(4):1057–1070 36597642 10.14245/ns.2244530.265 PMC9816586

[35] Zheng YC, Liu YT, Wei KC, Huang YC, Chen PY, Hsu YH et al (2022) Outcome predictors and clinical presentation of syringomyelia. Asian Journal of Surgery [Internet]. ;46(2):705–11. Available from: https://www.sciencedirect.com/science/article/pii/S1015958422008910 10.1016/j.asjsur.2022.06.150 35868963

[36] Lu C, Ma L, Yuan C, Cheng L, Wang X, Duan W et al (2022) Phenotypes and prognostic factors of syringomyelia in single-center patients with Chiari I malformation: moniliform type as a special configuration. Neurospine 19(3):816–827 36203304 10.14245/ns.2244332.166 PMC9537845

[37] Sun R, Pritsana Punyawai, Furtado N, Afshari FT, Gallo P (2025) Paediatric idiopathic syringomyelia — a follow-up of radiological and clinical outcomes into adulthood. Child S Nerv Syst. ;41(1):236 10.1007/s00381-025-06885-3 40670849

[38] Aliaga L, Hekman KE, Yassari R, Straus D, Luther G, Chen J et al (2011) A novel scoring system for assessing Chiari malformation type I treatment outcomes. Neurosurgery 70(3):656–665 10.1227/NEU.0b013e31823200a6 PMC6718293 21849925

[39] Guan J, Yuan C, Yao Q, Du Y, Fang Z, Zhang L et al (2023) A novel scoring system for assessing adult syringomyelia associated with CM I treatment outcomes. Acta neurologica Belgica [Internet]. ;123(3):807–14. Available from: https://pubmed.ncbi.nlm.nih.gov/37046133/10.1007/s13760-023-02264-4 37046133

[40] Yuan C, Du Y, Yao Q, Zhang C, Zhang L, Liu Z et al (2025) Natural history of Chiari I malformation-syringomyelia: longitudinal cohort study. J Neurol Neurosurg Psychiatry.jnnp-2025-336023. 10.1136/jnnp-2025-336023 PMC12703315 40675800

[41] Kim SH, Choi SW, Youm JY, Kwon HJ (2012) Syringo-subarachnoid-peritoneal shunt using T-tube for treatment of post-traumatic syringomyelia. J Korean Neurosurg Soc 52(1):58–58 22993681 10.3340/jkns.2012.52.1.58 PMC3440506

[42] Akakın A, Yılmaz B, Ekşi MŞ, Kılıç T (2015) Treatment of syringomyelia due to Chiari type I malformation with syringo-subarachnoid-peritoneal shunt. J Korean Neurosurg Soc 57(4):311 25932303 10.3340/jkns.2015.57.4.311 PMC4414780

[43] Agarwal A, Krishnamoorthy T (2010) Syringosubarachnoid shunt for syringomyelia associated with Chiari I malformation. Pediatr Radiol 40(S1):156–156 10.1007/s00247-010-1801-9 20725829

[44] Furst T, Jayden Allegakoen, Jalal MI, Singh R, Stone JJ (2024) Treatment of terminal syrinx with a syringo-subarachnoid shunt and the novel use of a titanium knot fastener: A case report. Cureus 10.7759/cureus.73440 PMC11633851 39664128

[45] Antkowiak L, Tabakow P (2021) Comparative assessment of three posterior fossa decompression techniques and evaluation of the evidence supporting the efficacy of syrinx shunting and filum terminale sectioning in Chiari malformation type I. A systematic review and network meta-analysis. World Neurosurg 152:31–43 34098134 10.1016/j.wneu.2021.05.124

[46] Perrini P, Anania Y, Cagnazzo F, Benedetto N, Morganti R, Carlo (2020) Radiological outcome after surgical treatment of syringomyelia-Chiari I complex in adults: a systematic review and meta-analysis. Neurosurg Rev 44(1):177–187 31953784 10.1007/s10143-020-01239-w

[47] Bogdanov EI, Faizutdinova AT, Mendelevich EG, Sozinov AS, Heiss JD (2018) Epidemiology of symptomatic Chiari malformation in Tatarstan: regional and ethnic differences in prevalence. Neurosurgery 84(5):1090–1097 10.1093/neuros/nyy175 PMC6505441 29788393

[48] Rothrock RJ, Lu VM, Levi AD (2021) Syrinx shunts for syringomyelia: a systematic review and meta-analysis of syringosubarachnoid, syringoperitoneal, and syringopleural shunting. Journal of neurosurgery Spine [Internet]. ;35(4):535–45. Available from: https://pubmed.ncbi.nlm.nih.gov/34330095/10.3171/2020.12.SPINE201826 34330095

[49] Zhou LN, Xiao X, Chen XY, Gu SX, Liu XD, Shou JJ et al (2024) The surgical strategy cerebrospinal fluid decompression facilitates outcomes of adults with Chiari malformation type I: an observational, real-world, single-center study of 528 patients. World Neurosurg 189:e841–e856 38986944 10.1016/j.wneu.2024.07.016

[50] Posterior Fossa Decompression With or Without Duraplasty for Chiari Type I Malformation With Syringomyelia [Internet] Clinicaltrials.gov. 2024 [cited 2025 Jul 25]. Available from: https://clinicaltrials.gov/study/NCT02669836

[51] Versus PFDRT in Chiari Decompression Surgery [Internet]. Clinicaltrials.gov. 2023 [cited 2025 Jul 25]. Available from: https://www.clinicaltrials.gov/study/NCT06079125

[52] Piper RJ, Afshari FT, Soon WC, Kolias AG, Dyson EW, Watkins L et al (2021) UK Chiari 1 study: protocol for a prospective, observational, multicentre study. BMJ Open 11(4):e043712 33846149 10.1136/bmjopen-2020-043712 PMC8048021

[53] Stoodley MA, Jones NR, Yang L, Brown CJ (2000) Mechanisms underlying the formation and enlargement of noncommunicating syringomyelia: experimental studies. Neurosurg Focus 8(3):1–7 16676925 10.3171/foc.2000.8.3.2

[54] Heiss JD, Snyder K, Peterson MM, Patronas NJ, Butman JA, Smith RK et al (2012) Pathophysiology of primary spinal syringomyelia. J Neurosurg Spine 17(5):367–380 22958075 10.3171/2012.8.SPINE111059 PMC3787878

[55] Thouvenin O, Keiser L, Cantaut-Belarif Y, Carbo-Tano M, Verweij F, Jurisch-Yaksi N et al (2020) Origin and role of the cerebrospinal fluid bidirectional flow in the central canal. eLife 9: e47699 10.7554/eLife.47699 31916933 10.7554/eLife.47699 PMC6989091

[56] Grimes DT, Boswell CW, Morante NFC, Henkelman RM, Burdine RD, Ciruna B (2016) Zebrafish models of idiopathic scoliosis link cerebrospinal fluid flow defects to spine curvature. Science 352(6291):1341–1344 27284198 10.1126/science.aaf6419 PMC5574193

[57] Yuan C, Zhang C, Wang J, Wu H, Chen Z, Jian F et al (2025) Spring;42(5):641–9 A novel minimally invasive surgical technique for posttraumatic syringomyelia: subarachnoid-subarachnoid bypass. Journal of neurosurgery Spine [Internet]. Available from: https://pubmed.ncbi.nlm.nih.gov/39983113/10.3171/2024.10.SPINE24987 39983113

[58] Yuan C, Xiong Z, Houyuan Lv, Ding C, Xia P, Xue H et al (2025) A Novel Surgical Technique for Post-traumatic Syringomyelia Progressing to the Medulla Oblongata: Evidence of Upward Drainage of Central Canal Fluid Within the Spinal Cord. Neurosurgery 10.1227/neu.0000000000003378 PMC12221135 40029068

