# Supplementary material for: Efficacy of neurosurgical intervention in syrinx resolution in patients presenting with Chiari malformation type I and syringomyelia: a systematic review and radiological meta-analysis
Source: Neurosurg Rev. 2025 Oct 20;48(1):724. doi: 10.1007/s10143-025-03864-9 (PMC12535944; doi:10.1007/s10143-025-03864-9)
Supplement: Supplementary file 1 — Supplementary Material 1 (PDF 134 KB) [file 10143_2025_3864_MOESM1_ESM.pdf]

Neurosurgery and syrinx resolution: search terms

Criterion 1: Chiari malformation type 1

MESH: "Chiari Malformation Type I with Syringomyelia" [Supplementary Concept]

Keywords: “Chiari Malformation Type I with Syringomyelia"[tw] OR “Chiari malformation type I”[tw] OR “Chiari malformation type i”[tw] OR “Arnold chiari malformation type 1”[tw] OR “Arnold chiari malformation type 1”[tw] OR “Arnold chiari malformation type i”[tw]

Final: **"Chiari Malformation Type I with Syringomyelia" [Supplementary Concept] OR “Chiari Malformation Type I with Syringomyelia"[tw] OR “Chiari malformation type I”[tw] OR “Chiari malformation type i”[tw] OR “Arnold chiari malformation type 1”[tw] OR “Arnold chiari malformation type I”[tw] OR “Arnold chiari malformation type i”[tw]**

Criterion 2: Neurosurgery

MESH: "Neurosurgical Procedures"[Mesh]

Keywords: “neurosurger*”[tw] OR “neurosurgical intervention*”[tw] OR “foramen magnum decompression*”[tw] OR “posterior fossa decompression*”[tw] OR “chiari malformation decompression*”[tw] OR “chiari malformation surger*”[tw] OR “suboccipital craniectom*”[tw] OR “decompression surger*”[tw]

Final: **"Neurosurgical Procedures"[Mesh] OR “neurosurger*”[tw] OR “neurosurgical intervention*”[tw] OR “foramen magnum decompression*”[tw] OR “posterior fossa decompression*”[tw] OR “chiari malformation decompression*”[tw] OR “chiari malformation surger*”[tw] OR “suboccipital craniectom*”[tw] OR “decompression surger*”[tw]**

Criterion 3: Syrinx

MESH: "Syringomyelia"[Mesh]

Keywords: “syrinx”[tw] OR “fluid-filled cyst”[tw] OR “syringomyelia cyst”[tw] OR “syringomyelia”[tw]

Final: **"Syringomyelia"[Mesh] OR “syrinx”[tw] OR “fluid-filled cyst”[tw] OR “syringomyelia cyst”[tw] OR “syringomyelia”[tw]**

Search number	Query	Sort By	Filters	Search Details	Results	Time
**4**	#1 AND #2 AND #3			("Chiari Malformation Type I with Syringomyelia"[Supplementary Concept] OR "Chiari Malformation Type I with Syringomyelia"[Text Word] OR "chiari malformation type i"[Text Word] OR "chiari malformation type i"[Text Word] OR "Arnold chiari malformation type 1"[Text Word] OR "arnold chiari malformation type i"[Text Word] OR "arnold chiari malformation type i"[Text Word]) AND ("Neurosurgical Procedures"[MeSH Terms] OR "neurosurger*"[Text Word] OR "neurosurgical intervention*"[Text Word] OR "foramen magnum decompression*"[Text Word] OR "posterior fossa decompression*"[Text Word] OR "chiari malformation decompression*"[Text Word] OR "chiari malformation surger*"[Text Word] OR "suboccipital craniectom*"[Text Word] OR "decompression surger*"[Text Word]) AND ("Syringomyelia"[MeSH Terms] OR "syrinx"[Text Word] OR "fluid-filled cyst"[Text Word] OR "syringomyelia cyst"[Text Word] OR "Syringomyelia"[Text Word])	243	14:45:04
**3**	"Syringomyelia"[Mesh] OR "syrinx"[tw] OR "fluid-filled cyst"[tw] OR "syringomyelia cyst"[tw] OR "syringomyelia"[tw]			"Syringomyelia"[MeSH Terms] OR "syrinx"[Text Word] OR "fluid-filled cyst"[Text Word] OR "syringomyelia cyst"[Text Word] OR "Syringomyelia"[Text Word]	6,406	14:43:19
**2**	"Neurosurgical Procedures"[Mesh] OR "neurosurger*"[tw] OR "neurosurgical intervention*"[tw] OR "foramen magnum decompression*"[tw] OR "posterior fossa decompression*"[tw] OR "chiari malformation decompression*"[tw] OR "chiari malformation surger*"[tw] OR "suboccipital craniectom*"[tw] OR "decompression surger*"[tw]			"Neurosurgical Procedures"[MeSH Terms] OR "neurosurger*"[Text Word] OR "neurosurgical intervention*"[Text Word] OR "foramen magnum decompression*"[Text Word] OR "posterior fossa decompression*"[Text Word] OR "chiari malformation decompression*"[Text Word] OR "chiari malformation surger*"[Text Word] OR "suboccipital craniectom*"[Text Word] OR "decompression surger*"[Text Word]	254,101	14:42:48
**1**	"Chiari Malformation Type I with Syringomyelia" [Supplementary Concept] OR "Chiari Malformation Type I with Syringomyelia"[tw] OR "Chiari malformation type I"[tw] OR "Chiari malformation type i"[tw] OR "Arnold chiari malformation type 1"[tw] OR "Arnold chiari malformation type I"[tw] OR "Arnold chiari malformation type i"[tw]			"Chiari Malformation Type I with Syringomyelia"[Supplementary Concept] OR "Chiari Malformation Type I with Syringomyelia"[Text Word] OR "chiari malformation type i"[Text Word] OR "chiari malformation type i"[Text Word] OR "Arnold chiari malformation type 1"[Text Word] OR "arnold chiari malformation type i"[Text Word] OR "arnold chiari malformation type i"[Text Word]	695	14:41:58
