# Supplementary figures and images for: Efficacy of neurosurgical intervention in syrinx resolution in patients presenting with Chiari malformation type I and syringomyelia: a systematic review and radiological meta-analysis
Source: Neurosurg Rev. 2025 Oct 20;48(1):724. doi: 10.1007/s10143-025-03864-9 (PMC12535944; doi:10.1007/s10143-025-03864-9)

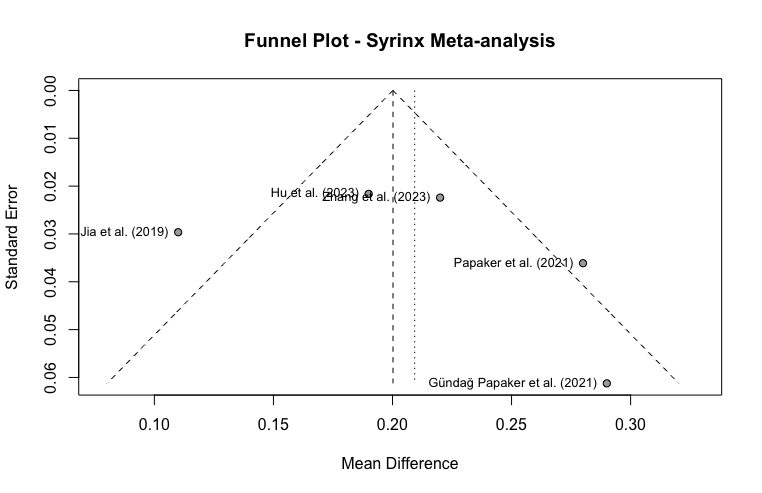


**Supplementary Figure 1.**

Supplement: Supplementary file 2 — Supplementary Material 2 (DOCX 62.8 KB) [file 10143_2025_3864_MOESM2_ESM.docx]

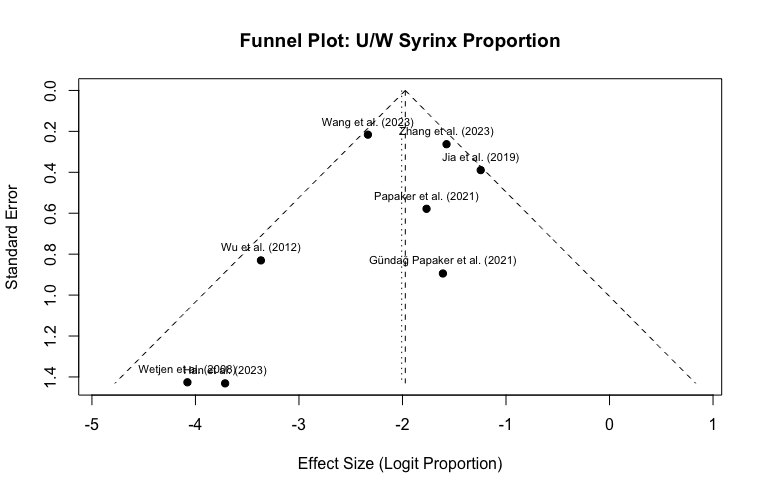


**Supplementary Figure 2.**

Supplement: Supplementary file 3 — Supplementary Material 3 (DOCX 65 KB) [file 10143_2025_3864_MOESM3_ESM.docx]

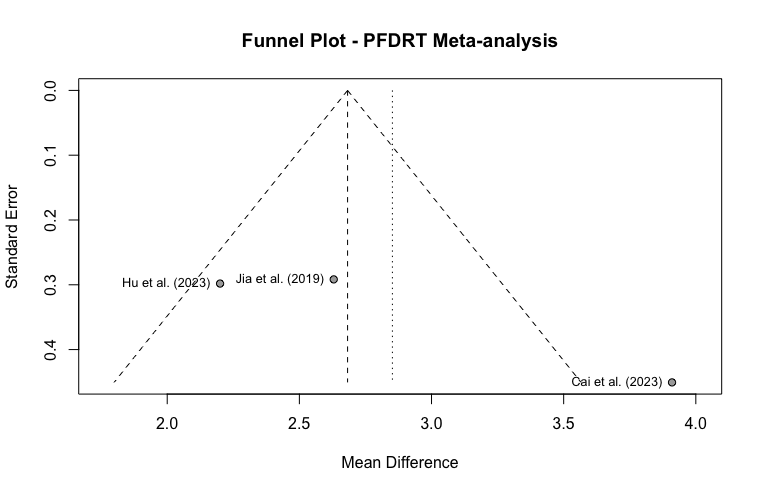


**Supplementary Figure 3.**

Supplement: Supplementary file 4 — Supplementary Material 4 (DOCX 55.8 KB) [file 10143_2025_3864_MOESM4_ESM.docx]

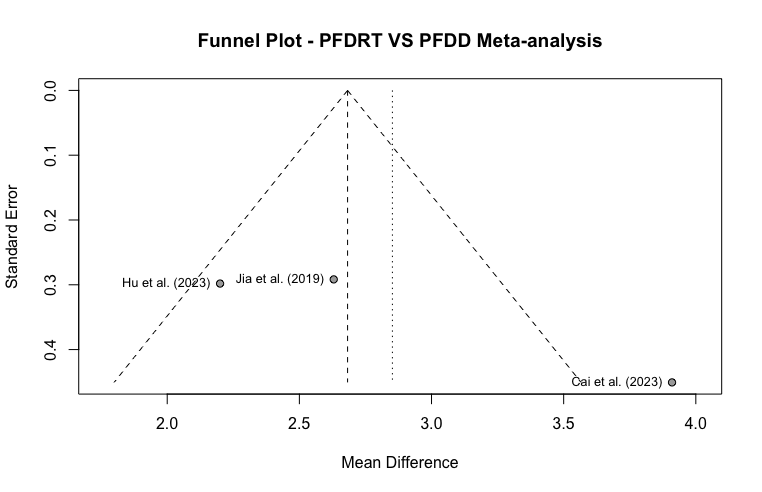


**Supplementary Figure 4.**

Supplement: Supplementary file 5 — Supplementary Material 5 (DOCX 56.9 KB) [file 10143_2025_3864_MOESM5_ESM.docx]

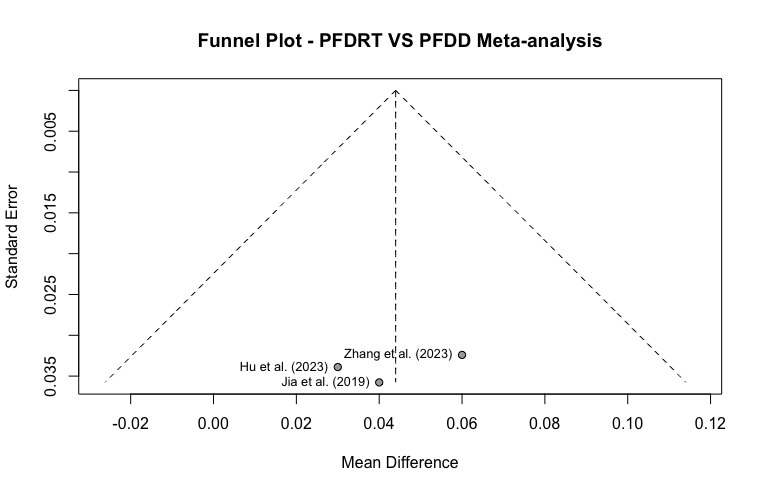


**Supplementary Figure 5.**

Supplement: Supplementary file 6 — Supplementary Material 6 (DOCX 58.8 KB) [file 10143_2025_3864_MOESM6_ESM.docx]

**Supplementary Figure 6.**


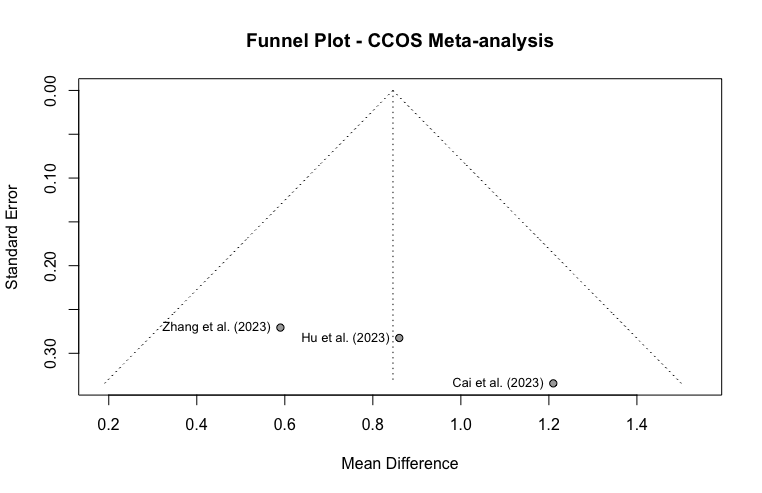

Supplement: Supplementary file 7 — Supplementary Material 7 (DOCX 54 KB) [file 10143_2025_3864_MOESM7_ESM.docx]
